# A systematic review and meta-analyses of interleukin-1 receptor associated kinase 3 (IRAK3) action on inflammation in *in vivo* models for the study of sepsis

**DOI:** 10.1371/journal.pone.0263968

**Published:** 2022-02-15

**Authors:** Trang H. Nguyen, Ilona Turek, Terri Meehan-Andrews, Anita Zacharias, Helen R. Irving

**Affiliations:** Department of Pharmacy and Biomedical Sciences, La Trobe Institute for Molecular Science, La Trobe University, Bendigo, Victoria, Australia; Holbaek Sygehus, DENMARK

## Abstract

**Background:**

Interleukin-1 receptor associated kinase 3 (IRAK3) is a critical modulator of inflammation and is associated with endotoxin tolerance and sepsis. Although IRAK3 is known as a negative regulator of inflammation, several studies have reported opposing functions, and the temporal actions of IRAK3 on inflammation remain unclear. A systematic review and meta-analyses were performed to investigate IRAK3 expression and its effects on inflammatory markers (TNF-α and IL-6) after one- or two-challenge interventions, which mimic the hyperinflammatory and immunosuppression phases of sepsis, respectively, using human or animal *in vivo* models.

**Methods:**

This systematic review and meta-analyses has been registered in the Open Science Framework (OSF) (Registration DOI: 10.17605/OSF.IO/V39UR). A systematic search was performed to identify *in vivo* studies reporting outcome measures of expression of IRAK3 and inflammatory markers. Meta-analyses were performed where sufficient data was available.

**Results:**

The search identified 7778 studies for screening. After screening titles, abstracts and full texts, a total of 49 studies were included in the systematic review. The review identified significant increase of IRAK3 mRNA and protein expression at different times in humans compared to rodents following one-challenge, whereas the increases of IL-6 and TNF-α protein expression in humans were similar to rodent *in vivo* models. Meta-analyses confirmed the inhibitory effect of IRAK3 on TNF-α mRNA and protein expression after two challenges.

**Conclusions:**

A negative correlation between IRAK3 and TNF-α expression in rodents following two challenges demonstrates the association of IRAK3 in the immunosuppression phase of sepsis. Species differences in underlying biology affect the translatability of immune responses of animal models to human, as shown by the dissimilarity in patterns of IRAK3 mRNA and protein expression between humans and rodents following one challenge that are further influenced by variations in experimental procedures.

## Introduction

Sepsis is one of the major causes of mortality in intensive care units, with approximately 49 million incident cases worldwide in 2017, and 11 million sepsis-related deaths that account for 19.7% of all global deaths [1–3]. Sepsis is defined as severe organ dysfunction or failure caused by dysregulated host inflammatory responses to microbial infections, and occurs as a complicated interplay of the pro-inflammatory and anti-inflammatory phases [1, 4]. Microbial infections activate innate immunity defences through the signalling pathways initiated by pattern recognition receptors including Toll-like receptors (TLRs) [5, 6]. Following the engagement of receptor ligands, TLRs are stimulated to induce the release of inflammatory cytokines. The adaptor Myeloid differentiation primary response protein 88 (MyD88) is recruited to TLRs, to form a myddosome complex with interleukin-1 receptor associated kinase (IRAK) family members including IRAK1, IRAK2 and IRAK4 [5, 7]. IRAK4 activates IRAK1 or IRAK2 which dissociate from the complex and interact with tumour necrosis factor (TNF) receptor associated factor 6 (TRAF6) [8]. TRAF6 triggers activation of nuclear factor kappa-light-chain-enhancer of activated B cells (NF-κB) transcription factor which induces transcription of inflammatory cytokine genes such as TNF-α and IL-6 [5]. IRAK3 is mainly found in monocytes and macrophages, hence it is also known as IRAK-M [9–11]. IRAK3 inhibits the dissociation of IRAK1 or IRAK2 from myddosome complexes, thus disables the interaction of IRAK1 or IRAK2 with TRAF6, leading to downregulation of NF-κB activation [9, 12]. Thus, IRAK3 plays a crucial role in modulating TLR signalling pathways of innate immunity. Part of the molecular mechanism of action of IRAK3 involves interactions with the death domain of these proteins [11, 13], and potentially a guanylate cyclase centre that is found in its pseudokinase domain, and able to produce cyclic guanosine monophosphate [14].

TNF-α and IL-6 are critical pro-inflammatory cytokines in sepsis due to their consistent association with the severity, mortality and organ dysfunction of patients with sepsis [1, 6, 15–17]. IL-6 belongs to the cytokine network that regulates the acute phase of response to inflammation in sepsis [18, 19]. Serum levels of IL-6 and TNF-α are highly elevated in newborns with sepsis compared to healthy newborns, and lower in the survivors of elderly patients with sepsis than non-survivors, indicating their relation to inflammatory syndrome of sepsis [16, 17].

The initial phase of sepsis is characterized by hyper-inflammatory responses, also known as a “cytokine storm”, with uncontrolled expression of pro-inflammatory cytokines [1]. However, about 70% of patients who died from sepsis within three days or several weeks after the initial onset of the disorder, showed marked immunosuppression with decreased cytokine production [1]. The second phase of sepsis–immunosuppression or anti-inflammatory phase–occurs simultaneously with the pro-inflammatory phase, although hyper-inflammatory responses are predominant in the initial period of sepsis [4]. Deaths occurring during the immunosuppression phase are frequently caused by the inability of patients to control the primary infection or acquisition of secondary opportunistic pathogen infections [1, 4].

The immunosuppression during sepsis is associated with an upregulation of IRAK3 mRNA and protein expression [20]. A positive association between IRAK3 single nucleotide polymorphisms and sepsis susceptibility was reported in a Han Chinese population [21]. IRAK3 transcript expression is markedly induced in clinical samples of septic patients compared to healthy subjects, and highly correlated to severity and mortality of sepsis [2, 20–22]. Additionally, sepsis non-survivors displayed higher IRAK3 mRNA expression than the survivors [20, 22]. IRAK3 is associated with the immunosuppression phase of sepsis and also endotoxin tolerance that is one mechanism underlying the immunosuppression [9, 20, 23–27]. During endotoxin tolerance, cells or organisms exposed to endotoxins, such as lipopolysaccharide (LPS, an outer membrane component of Gram-negative bacteria), are unable to respond to further endotoxin stimulation [28]. IRAK3 deficiency causes an elevation in inflammatory cytokine production compared to wild type following LPS re-challenge, indicating its importance during endotoxin tolerance [9, 29, 30].

The effect of IRAK3 in sepsis has been investigated widely using cell culture [26, 31–33], clinical samples [20, 21, 34, 35], and *in vivo* animal [32, 36–44] and human [45] models. However, the mechanism of IRAK3 action on sepsis is not fully understood. IRAK3 exerts its specific function(s) in signalling pathways that depend on type of pathogenesis, duration of challenge and type of stimuli. For instance, IRAK3 markedly inhibits cytokine (TNF-α, IL-6, IL-1β) mRNA or protein expression in septic mice challenged with LPS or bacteria [36, 38, 39]. However, in several studies reporting results of *in vivo* challenge with bacteria in mice, IRAK3 knockout did not demonstrate significant effects on inflammatory cytokine production [40, 41]. A previous systematic review and meta-analysis examining IRAK3 function on inflammation in *in vitro* cell studies confirmed subtle effects of IRAK3 expression in down-regulating immune responses at a cellular level [46]. However, to our knowledge, a systematic review or meta-analysis of IRAK3 effects in *in vivo* models for the study of sepsis has not been conducted to date. Thus, the purpose of this review is to analyse IRAK3 expression, and its effects and correlation to inflammatory cytokine expression (TNF-α and IL-6), using meta-analyses of quantitative data from previous studies of *in vivo* animal or human models after one- or two-challenge interventions which mimic sepsis conditions. One-challenge interventions, where the subjects (e.g., mice) were stimulated with endotoxin (e.g., LPS) or bacteria or underwent other immunostimulatory procedures, can be used to study pathophysiology of sepsis. Two-challenge interventions can be used to mimic the immunosuppression phase of sepsis. At specific periods following one- or two-challenge interventions, the outcomes of interest–IRAK3, IL-6 and TNF-α expression levels–were measured.

## Materials and methods

### Search strategy and identification of studies

This systematic review and meta-analyses have been registered in the Open Science Framework (OSF) (Registration DOI: 10.17605/OSF.IO/V39UR). Six databases (Scopus, Web of Knowledge, Medline, ScienceDirect, Embase, PubMed) were searched systematically from the earliest date available until 18 November 2021. The search keywords were conducted using three concepts: IRAK, endotoxin tolerance, and sepsis. Synonyms within each concept were combined using the OR Boolean operator (IRAK or Interleukin-1 receptor associated kinase, endotoxin tolerance or lipopolysaccharide tolerance).

Titles and abstracts were screened initially by one reviewer (T.H.N.) using selection criteria described in Table 1. Selected studies from the first screening were then screened independently by two reviewers (T.H.N. and H.R.I./I.T./T.M.-A.). The full text of the studies was verified independently by two reviewers (T.H.N. and H.R.I./I.T./T.M.-A.) using the same selection criteria. The reviewers discussed differences in opinion until consensus was reached. Citation tracking and reference checking of the included articles was also performed.

**Table 1:** Study selection criteria.

Criteria
Must contain the concept of inflammation, regulation or intervention of inflammation.
Must contain the concept of IRAK/lipopolysaccharide/endotoxin tolerance/sepsis.
Must report data obtained from mammalian species.
Must be peer-reviewed research.
Must use *in vivo* models.
Must contain at least one of the outcomes (IRAK3 mRNA and protein expression, TNF-α mRNA expression and protein level, IL-6 protein level).
Must be in English.

### Population

The included studies were restricted to human and animal *in vivo* studies (Table 2).

**Table 2:** Basis for comparisons by meta-analyses.

Population	Durations of outcome measurement after intervention	Comparison groups	Outcome measures
Humans	Short term (ST) (1h – 3h)	Control versus one-challenge	IRAK3 mRNA expression
	Intermediate term (IT) (4h – 15h)	One-challenge versus two-challenges	IRAK3 protein expression
	Long term (LT) (16h – 72h)		TNF-α mRNA and protein level
			IL-6 protein level
Rodents (Mice and rats)	Short term (ST) (1h – 3h)	Control versus one-challenge	IRAK3 mRNA expression
Sheep	Intermediate term (IT) (4h – 15h)	One-challenge versus two-challenges	IRAK3 protein expression
Cows	Long term (LT) (16h – 72h)	IRAK3 knockout versus IRAK3 present	TNF-α mRNA and protein level
Pigs			IL-6 protein level

### Interventions

Healthy human volunteers were administered LPS (a TLR4 agonist). Animals were *in vivo* challenged with bacteria (*Neisseria meningitidis*, *Klebsiella pneumoniae*, *Streptococcus pneumoniae*, *Haemophilus influenzae*, *Escherichia coli*) or inflammation-inducing chemicals (e.g., TLR4 agonist–LPS, TLR2 agonists–Pam3CSK4 (Pam3CysSerLys4) and lipoteichoic acid, TLR7 agonist– 1V136 or R848, TLR3 agonist–pIC (polyinosinic: polycytidylic acid), or TLR9 agonists–CpG DNA and ISS-ODN (immunostimulatory sequence oligodeoxynucleotides)). Alternatively, mice underwent cecal ligation puncture (CLP) procedure, in which the cecum is punctured to allow the release of fecal material into the peritoneal cavity [47]; or were injected with cecal slurry to generate polymicrobial infection; or using experimental colitis-induced model by drinking 5% DSS solution for 3d or 5d prior to outcome measurement.

In two-challenge intervention, the subjects were first treated with one-challenge intervention following which the subjects were treated again with endotoxin or bacteria, or the samples (e.g., cells, plasma) were extracted from these subjects and *ex vivo* treated with endotoxin or bacteria.

### Comparisons

There are three types of comparisons: 1) comparisons between a control group which was treated with phosphate-buffered saline (PBS) or sterile water or underwent sham procedure (the cecum was exteriorized but neither ligated nor punctured, as a control for mouse CLP model; or injected with glycerol-PBS as a control for the cecal slurry-treated mouse; or drinking water only, as a control for mouse colitis model) and a group receiving one-challenge as described above; 2) comparisons between a group receiving one-challenge and a group receiving two-challenges as described above; and 3) comparisons between an IRAK3 silencing/knockout group and an IRAK3 wild type group of mouse *in vivo* models.

### Outcomes

Outcomes that are measures of IRAK3 and inflammatory cytokines (TNF-α, IL-6) mRNA and protein expression level were included.

### Research design

The articles included must be research studies reporting *in vivo* intervention. Review articles and *in vitro* studies were excluded.

### Data extraction

Data including species (human or animal), study design, description of intervention, intervention duration and outcome measure were extracted from each included study (S1 Table). Data extraction was performed by one author (T.H.N.) and verified by a second author (either H.R.I., T.M.-A. or I.T.). In cases where there were no numerical data provided in the articles, the data were estimated from graphs. If data with insufficient transparency were reported in the articles, data were requested from the original author or qualitatively assessed if not provided. The relevant data were extracted and entered directly into Review Manager (RevMan) Version 5.3 by one author (T.H.N.) and checked by a second author (H.R.I./T.M.-A./I.T./A.Z.).

### Quality analysis

The methodological quality of included animal studies was assessed according to the Systematic Review Centre for Laboratory Animal Experimentation (SYRCLE) risk of bias tool [48]. SYRCLE was adapted from the Cochrane risk of bias tool for animal intervention studies. SYRCLE’s risk of bias tool has a total of 10 items, which were scored as low, high, or unclear risk of bias to assess selection, performance, detection, attrition, and reporting biases. Articles were not excluded from this review on the basis of quality.

A suitable risk of bias tool for the human *in vivo* studies could not be identified and therefore the methodological quality assessment of human studies could not be performed.

### Data synthesis

Where possible, studies were grouped for meta-analyses based on population, intervention and outcome measures of interest (Table 2). Meta-analyses for human studies were separated from meta-analyses of animal studies; meta-analyses were also separated from different animal species studies (rodents, pigs, sheep, cows). Moreover, interventions of challenges with bacteria, or bacteria-derived compounds (TLR4 agonist–LPS, TLR2 agonist–Pam3CSK4, TLR2 agonist–lipoteichoic acid) or undergoing polymicrobial CLP procedure are grouped in a meta-analysis; and those using other challenge chemicals (e.g., TLR3 agonist, TLR7 agonist, TLR9 agonist) are in separate meta-analyses. RevMan 5.3 software was used to perform the meta-analyses. Standardized mean difference (SMD) and 95% confidence intervals (CIs) were calculated to indicate the effect size for outcome measures of interest. The I^2^ value was used for assessing heterogeneity [49]. A value of 0% was interpreted as indicating no observed heterogeneity, and a value of 100% was considered to be a completely heterogeneous sample. Values of 25%, 50% and 75% indicated low, moderate and high levels of heterogeneity [49].

## Results

### Yield

**Fig 1:**
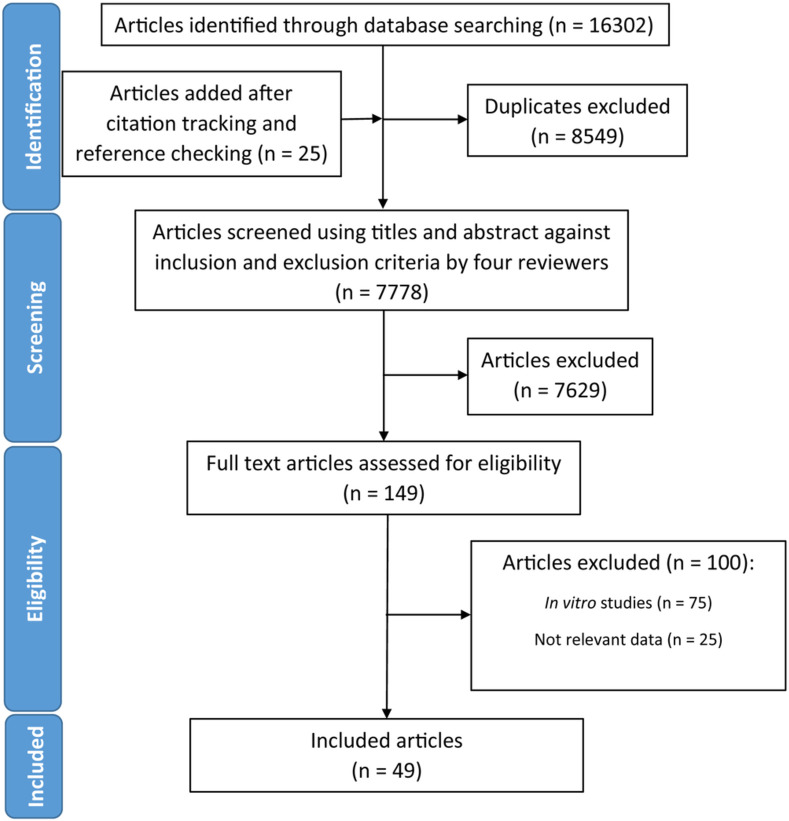
PRISMA flowchart. PRISMA flowchart depicting the process of search and selection of studies for the systematic review.

A total of 16302 studies were identified through database searches, and 8549 duplicate studies that appeared in more than one database were removed (Fig 1). Citation tracking and reference checking added an additional 25 studies (Fig 1). The studies underwent screening of titles and abstracts based on study selection criteria (Table 1), and as a result 149 studies were selected (Fig 1). After full-text screening, 49 studies were included in the systematic review as shown in S1 Table. Studies excluded from full-text screening and the reasons for exclusion can be found in S2 Table.

### Characteristics of included studies

Of the 49 included *in vivo* studies, 12 studies reported data from experiments performed using *in vivo* human model [45, 50–60], 34 studies [29, 32, 36–41, 43, 61–85] used rodents (mice or rats), and the remaining three used sheep [44], cows [86] or pigs [42]. All included human studies (12 studies) used LPS administration with *in vivo* doses of 2–4 ng/kg. Most animal studies (16 studies) used LPS administration with *in vivo* doses of 0.1–45 mg/kg in mouse or 2–400 μg/mouse via injection or 100 μg/mL via inhalation in mouse or 10 mg/lamb/injection, and with *ex vivo* doses of 1–1000 μg/mL; eight used treatments with bacteria (6000–10^7^ colony-forming units (CFUs) of *N*. *meningitidis*, *K*. *pneumoniae*, *S*. *pneumoniae*, *H*. *influenzae*, or *E*. *coli*) as a challenge [32, 36, 40–42, 65, 79, 86]; six used CLP model [36, 38, 39, 76, 78, 82]; one used cecal slurry injection [83]; three studies used experimental colitis model [67, 68, 84]; one animal study used lipoteichoic acid [61]; one used 10–40 μg/mouse CpG DNA (TLR9 agonist) [63]; two used 500 nmole 1V136 or 20–100 μg R848 (TLR7 agonists), 25 μg/mouse Pam3CSK4 (TLR2 agonist), or 25 μg/mouse pIC (TLR3 agonist) [62, 81].

Of included studies, there were 20 studies (12 mouse studies and 8 human studies) in which two-challenge intervention was performed [29, 36, 38, 39, 42–45, 51, 52, 54, 55, 58–64, 86]; and nine studies made use of IRAK3 gene knockout or silencing [32, 36–41, 67, 68]. Most studies (44 studies) measured outcomes at intervals ranging between 1h and 72h following the first challenge, while several studies measured outcomes at 5d [43], 7d [44, 59, 67, 68], 10d [86], or 14d [42]. After the second challenge, outcomes of interest were measured between 2h and 24h. A previous systematic review of *in vitro* cell studies classified the intervals of outcome measured after challenge as short term (ST, 1h – 3h), intermediate term (IT, 4h – 15h), or long term (LT, 16h – 48h) [46]. Human and mouse *in vivo* models observed significant increases of TNF-α and IL-6 levels at ST (1h to 3h), and IRAK3 mRNA expression at IT (4h and 6h) after one-challenge [38, 41, 45]. Also, IRAK3 was reported to have significant effect on TNF-α mRNA and protein expressions at 6h and 24h following *in vivo* challenge of mice with bacteria [32, 36]. As there is similarity in important intervals between *in vitro* and *in vivo* models of challenge, and for ease of comparison between *in vitro* and *in vivo* models, the intervals in this systematic review of *in vivo* studies are classified as short term (ST, 1h – 3h), intermediate term (IT, 4h – 15h), and long term (LT, 16h – 72h).

### Quality

For items 1, 3, 4, 5, 6, and 7 in the SYRCLE’s risk of bias tool [48], the majority of *in vivo* animal studies had an unclear risk of bias because relevant information in the publications was not reported (S3 Table). Of 37 animal studies, 11 studies did not account for missing data and therefore were rated as having an unclear risk of bias for item 8 (S3 Table). The quality assessment of included human *in vivo* studies could not be performed due to the lack of a suitable tool.

### Intervention: One challenge

#### Effect of one-challenge intervention on IRAK3 mRNA and protein expression

**Fig 2:**
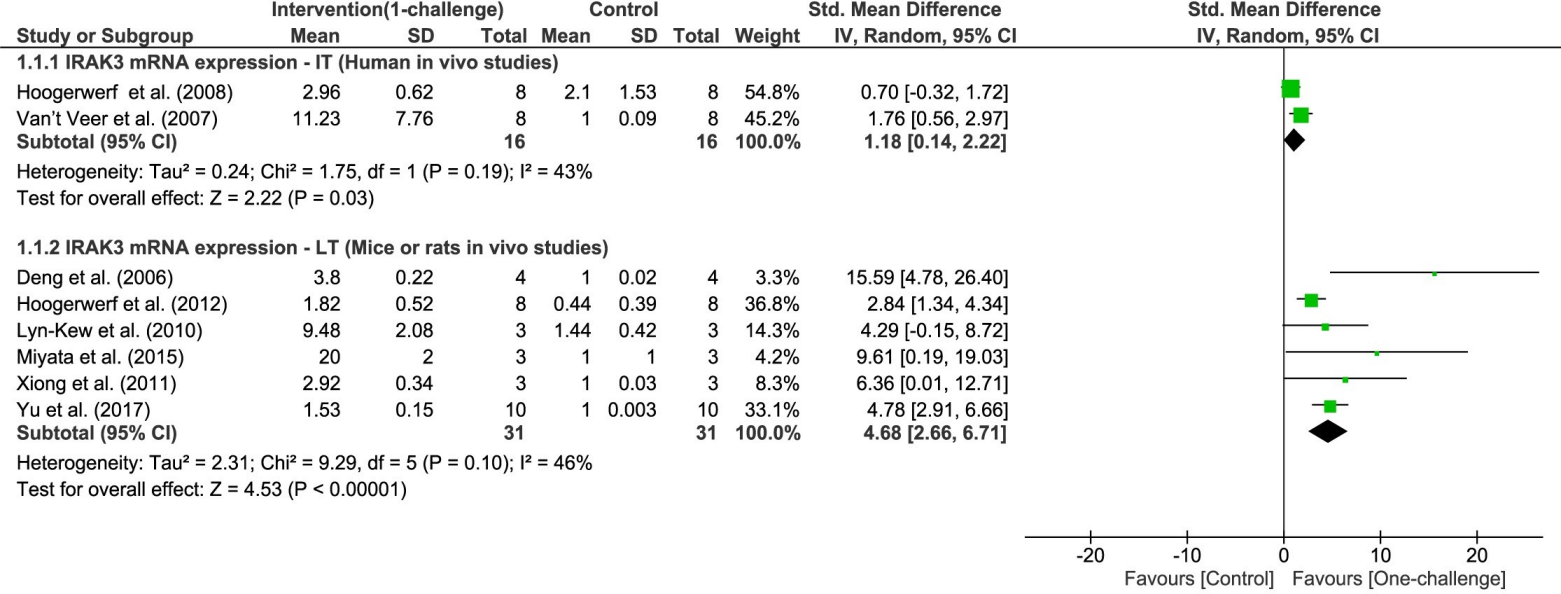
IRAK3 mRNA expression outcome: One-challenge intervention group versus control group. Two independent meta-analyses were conducted for human studies (1.1.1) and rodent studies (1.1.2). In human *in vivo* studies, the subjects were administered with LPS via intravenous injection [45] or instillation into the contralateral lung [56], and IRAK3 mRNA expression in whole blood [45] or alveolar macrophages [56] was measured. Mice or rats underwent CLP [36, 38] or were inoculated intranasally with *K*. *pneumoniae* [40], or inoculated or injected with LPS [29, 43], and IRAK3 mRNA expression was measured in samples taken from peritoneum [29], lung [36, 38, 40], or eye [43] at long term (LT; 16h – 72h).

Two independent meta-analyses of IRAK3 mRNA expression were performed for human or rodent studies (Fig 2). Meta-analysis of IRAK3 mRNA expression in human *in vivo* studies shows significant increase at IT following administration with LPS (two studies, P = 0.03, I^2^ = 43%, Fig 2). The fluctuations of IRAK3 mRNA expression in humans were significantly induced at 2h (ST), and reached maximum level at 4h and 6h (IT), followed by decreases at 8h (IT) and 24h (LT) after LPS administration [45]. Additionally, IRAK3 protein expression reached peak at 2-3h (ST), and decreased to baseline at 24h (LT) [45].

Meta-analysis of IRAK3 mRNA expression in rodent models identified significant increases at LT following *in vivo* challenge using CLP procedure, bacteria or LPS (six studies, P < 0.00001, I^2^ = 46%, Fig 2). IRAK3 mRNA expression markedly increased in lung macrophages or homogenates, intestinal mucosa and liver from mice between ST and LT [38, 40, 70, 71], reached peak at 24h (LT) following one-challenge with CLP/ LPS/ *K*. *pneumoniae* [38]. In rats, the increase of IRAK3 mRNA expression in eye tissue was reported at 5d following one-challenge with LPS [43]. Therefore, in rodents IRAK3 mRNA expression increases at ST and IT to reach peak level at LT and remains stable for up to 5d following *in vivo* challenge [38, 40, 43, 71]. While IRAK3 mRNA expression in cow mammary tissue significantly increased at LT (24h) [86], IRAK3 mRNA decreased at ST (3h) in pig spleen after *in vivo* challenge with *E*. *coli* [42]. These results suggest that tissue / cell types and species influence IRAK3 expression.

Meta-analysis of IRAK3 protein expression could not be conducted as the studies differed in their interventions. In single rodent *in vivo* studies, IRAK3 protein expression was reported to markedly increase at 3h (ST) in hepatic tissue following challenge with LPS [64], and at 24h (LT) in Kupffer cells (specialized liver cells) of mice injected with CpG DNA [63], but did not significantly change at 5d after challenge with LPS in the iris–ciliary body of eyes of rats [43]. These results therefore suggest that an increase of IRAK3 protein expression also embarks at ST in both human and rodent after *in vivo* one-challenge. However, the length of time in which IRAK3 levels remain significantly higher compared to baseline in rodents is longer than in humans.

#### Effect of one-challenge intervention on inflammatory cytokine expression

**Fig 3:**
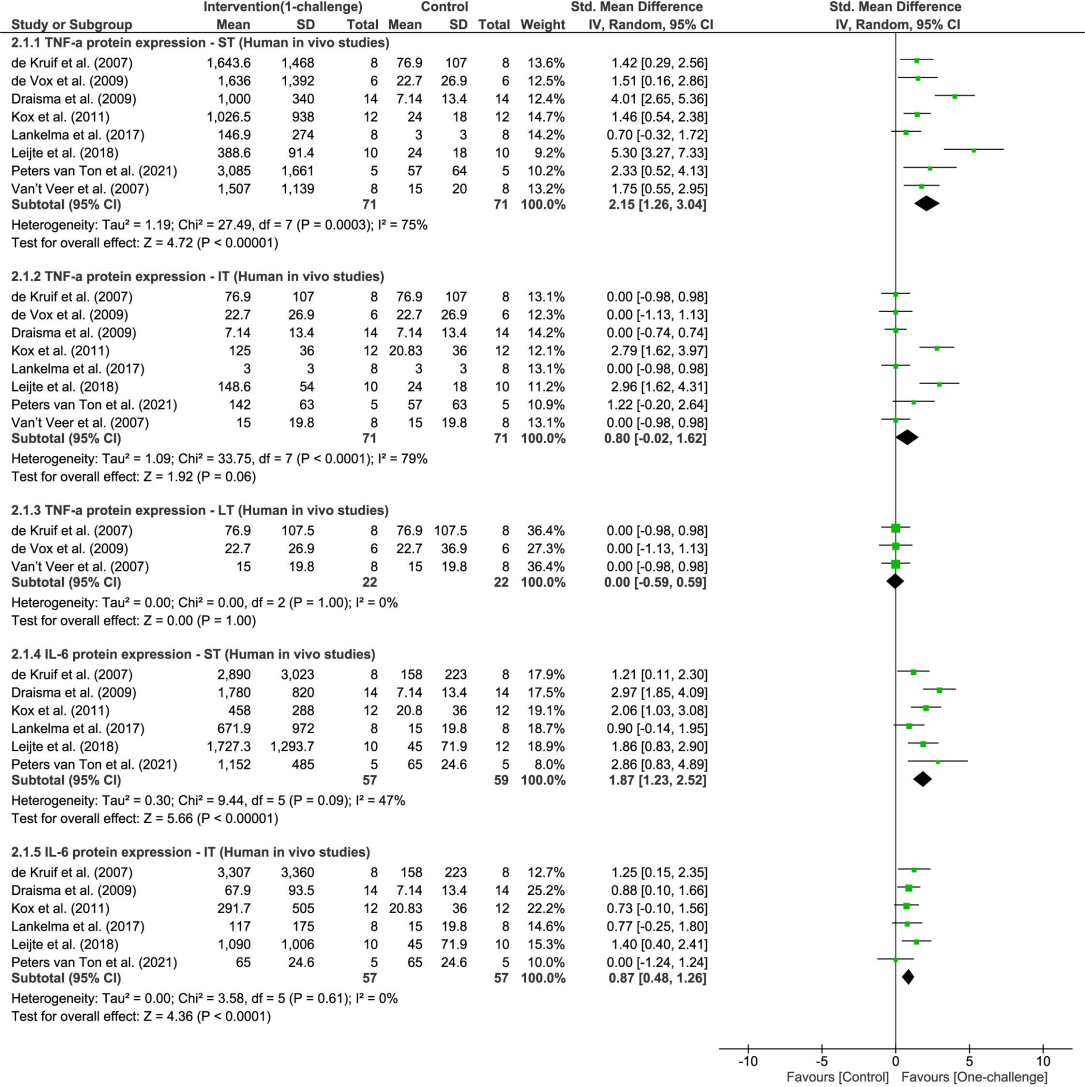
TNF-α and IL-6 protein expression outcome in humans: One-challenge intervention group versus control group. In one-challenge intervention, human subjects were administered with LPS [45, 50–52, 54, 55, 57, 60]. TNF-α and IL-6 protein level was measured at short term (ST; 1h – 3h), intermediate term (IT; 4h – 15h), or at long term (LT; 16h – 72h).

Meta-analyses of human *in vivo* models show significant increase of TNF-α protein level only at ST (eight studies, P < 0.00001, I^2^ = 75%, Fig 3), and increased IL-6 level at both ST and IT after one-challenge with LPS (ST, six studies, P < 0.00001, I^2^ = 47%; IT, six studies, P < 0.0001, I^2^ = 0%, Fig 3). TNF-α and IL-6 levels reached maximum levels between 1h and 3h (ST) in human blood, followed by significant decreases between 4h and 24h (IT and LT) post-treatment [45, 50, 53–55, 57, 87, 88] (Fig 3). Two single studies reporting TNF-α and IL-6 protein levels significantly increased in humans at 1.5h and 2h (ST) after *in vivo* challenge with LPS, could not be included in the meta-analysis due to presentation of data in different formats [53, 58].

Meta-analyses of *in vivo* one-challenge in rodents indicate significant elevation of TNF-α and IL-6 at ST, IT and LT (meta-analyses of TNF-α: ST, five studies, P = 0.002, I^2^ = 87%, IT, seven studies, P = 0.003, I^2^ = 93%, LT, 14 studies, P < 0.00001, I^2^ = 93%; meta-analyses of IL-6: ST, six studies, P < 0.00001, I^2^ = 41%, IT, five studies, P < 0.0001, I^2^ = 72%, LT, ten studies, P < 0.00001, I^2^ = 91%, S1 and S2 Figs). Two single studies not included in the meta-analysis due to differing challenge agents, reported TNF-α protein level significantly increases at 2h and 3h (ST) in mice challenged with R848, 1V136, Pam3CSK4, pIC or ISS-ODN [62, 81]. In addition, TNF-α protein expression reached peak levels between 1h and 3h (ST) in lung homogenates, serum or blood of mice after one challenge with *N*. *meningitidis* or *K*. *pneumoniae* or LPS [65, 66, 70, 72], and in serum of pigs injected with *E*. *coli* [42]. Thus, in rodent *in vivo* models, there is a rapid onset of TNF-α and IL-6 cytokine production at ST as seen in humans *in vivo* models [50, 54, 57, 65, 66]. However, TNF-α protein expression in humans decreased to baseline more promptly than in rodents following *in vivo* one-challenge (Figs 3 and S1).

#### Effect of IRAK3 on inflammatory cytokine expression following one-challenge in mice *in vivo* studies

**Fig 4:**
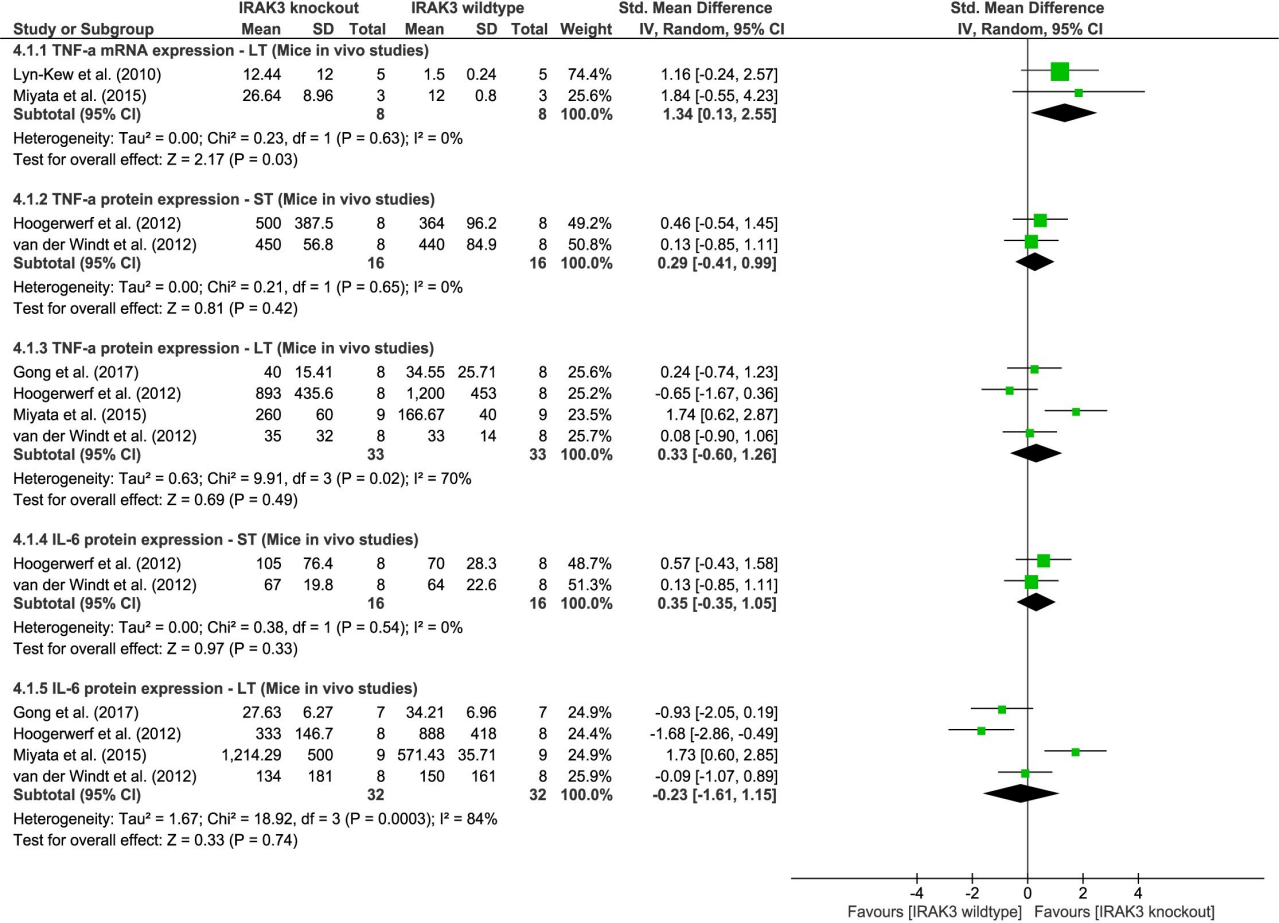
TNF-α and IL-6 mRNA or protein expression outcome in mice: IRAK3 knockout group versus IRAK3 wildtype group after one-challenge intervention. In one-challenge intervention, mice inhaled LPS [37], or were inoculated with *H*. *influenzae* [32], or *K*. *pneumoniae* [41], or *S*. *pneumoniae* [40]. TNF-α or IL-6 mRNA or protein expression was measured at short term (ST; 1h – 3h) or long term (LT; 16h - 72h) after one-challenge.

Several mouse *in vivo* studies used IRAK3 gene knockout or silencing to examine effects of IRAK3 on cytokine production. Meta-analysis identifies a significant effect of IRAK3 in inhibiting TNF-α mRNA expression in mice at LT after *in vivo* one-challenge intervention (two studies, P = 0.03, I^2^ = 0%, Fig 4). However, additional meta-analyses suggest no effect of IRAK3 on protein expression of TNF-α or IL-6 at ST or LT (meta-analyses of TNF-α: ST, two studies, P = 0.42, I^2^ = 0%, LT, four studies, P = 0.49, I^2^ = 70%; meta-analyses of IL-6: two studies, P = 0.33, I^2^ = 0%, LT, four studies, P = 0.74, I^2^ = 84%, Fig 4). Only one included study reported an inhibitory effect of IRAK3 on TNF-α and IL-6 protein production in mice following *in vivo* one-challenge with 5 × 10^7^–1 × 10^8^ CFU *H*. *influenza* inoculation [32]. Other *in vivo* mouse studies reported no effect of IRAK3 on inflammatory cytokines in mice challenged with bacteria or LPS, but their experimental designs varied in administration of immunomodulatory agent, including intranasal inoculation with 1 × 10^4^ CFU of *K*. *pneumoniae* [40], intranasal inoculation of 5 × 10^4^ or intravenous infection with 5 × 10^5^ CFU of *S*. *pneumoniae* [41], or LPS inhalation [37]. In addition, Hoogerwerf *et al*. [40] reported higher protein levels of TNF-α in IRAK3 knockout cells after *in vitro* stimulation with bacteria or LPS than wild type cells, which was contrary to their findings from *in vivo* mouse models, and they suggested that bacteria burden over-ruled the IRAK3 effect. On the other hand, IRAK3 knockout mice stimulated using colitis-induced model show significantly increased mRNA and protein expression of TNF-α and IL-6 compared to IRAK3 wild type mice [67, 68].

### Intervention: Two challenges

#### Effect of two-challenge intervention on expression of IRAK3

**Fig 5:**
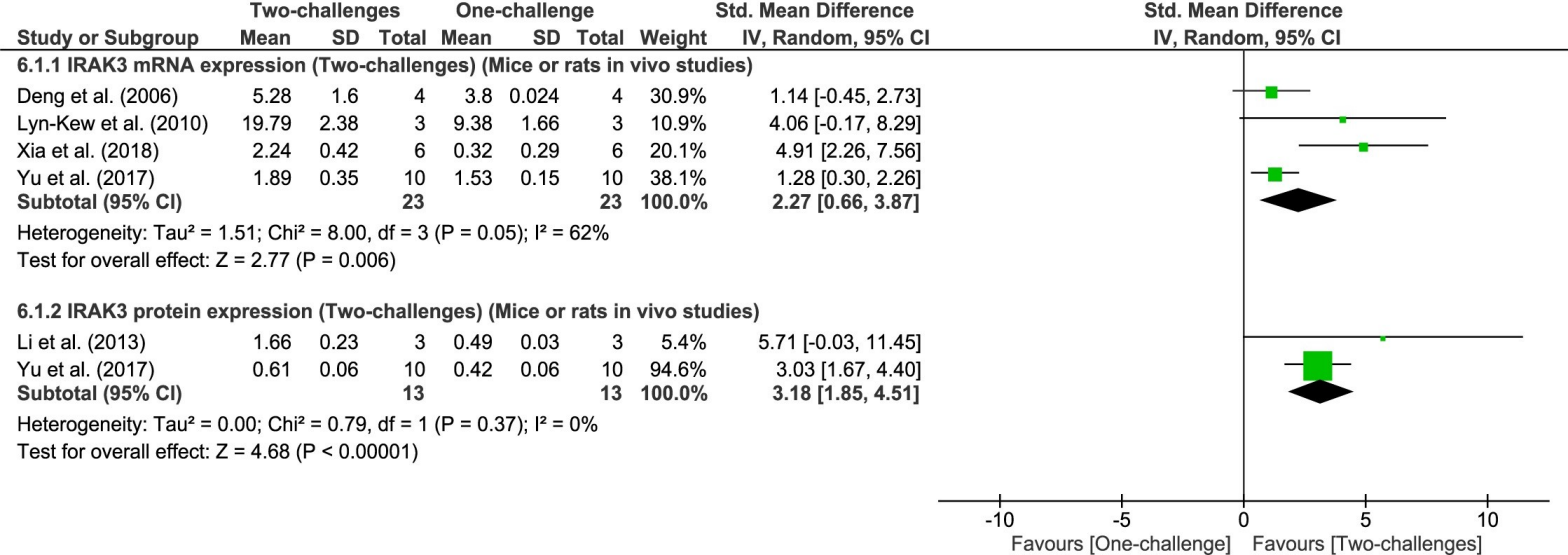
IRAK3 mRNA and protein expression outcome: One-challenge intervention group versus two-challenge intervention group. Mice underwent CLP or sham procedure; at 24h (LT) after CLP sham macrophages or monocytes were extracted and *ex vivo* treated with LPS. At 4h (IT) [38], 6h (IT) [36] and 24h (LT) [39] post-*ex vivo* treatment outcomes were measured. In one-challenge intervention, IRAK3 mRNA or protein expression was measured in cells extracted from sham-treated mice, and then *ex vivo* treated with LPS. In two-challenge intervention, IRAK3 mRNA or protein expression was measured in cells extracted from CLP-treated mice, and *ex vivo* treated with LPS. Alternatively, mice or rats were injected with LPS; at 24h (LT) after first LPS challenge mice or rats were treated again with LPS; and following the second challenge at 3h (ST) [64], at 24h (LT) [43] IRAK3 mRNA or protein expression in hepatic tissues [64] or enucleated eyes [43] were measured. In one-challenge intervention group, mice or rat were pre-treated with phosphate buffered saline or sterilized physiological saline for 24h [64] or 5d [43], and then treated with LPS, for 3h [64] or 24h [43] when outcomes were measured.

No human *in vivo* studies investigating IRAK3 expression following two challenges were identified in our systematic search. Meta-analyses of rodent *in vivo* studies indicate significant increases of IRAK3 mRNA and protein expression following two challenges with LPS or two different challenges (e.g., first challenge using CLP and the second challenge performed *ex vivo* with LPS) compared to one challenge (meta-analysis of IRAK3 mRNA expression: four studies, P = 0.006, I^2^ = 62%; meta-analysis of IRAK3 protein expression: two studies, P < 0.00001, I^2^ = 0%, Fig 5). The increase in IRAK3 mRNA and protein expression was observed between 3h (ST) and 24h (LT) following the second challenge (Fig 5). Similarly, IRAK3 mRNA expression in spleen tissue of pigs fed with diet supplemented with *L*. *acidophilus* for 14 days and challenged with *E*. *coli* for 3h via oral administration, is significantly elevated compared to pigs fed with 14-day-basal diet (without *L*. *acidophilus*) or challenged once with *E*. *coli* [42]. Although *in vivo* one challenge with *E*. *coli* via intramammary treatment up-regulated IRAK3 mRNA expression in cow mammary tissue, IRAK3 mRNA expression was not enhanced following two-challenges with LPS or *E*. *coli* [86], suggesting the effect of challenges on IRAK3 expression may depend on tissue or cell types and mode of administration.

#### Effect of two-challenge intervention on expression of inflammatory cytokines

**Fig 6:**
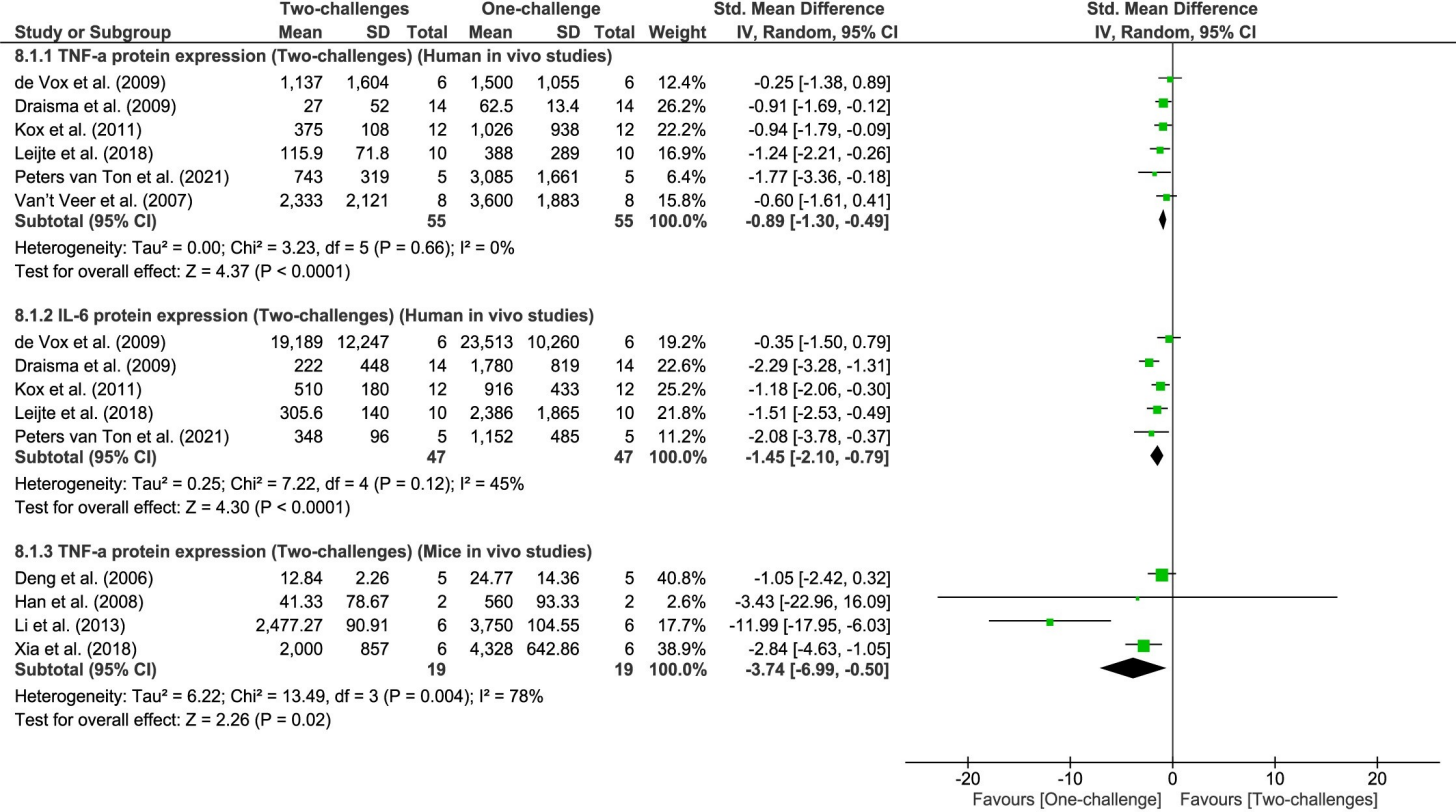
TNF-α and IL-6 protein expression outcome: One-challenge intervention group versus two-challenge intervention group. Independent meta-analyses of TNF-α and IL-6 protein expression were conducted for human studies (8.1.1 and 8.1.2) or rodent studies (8.1.3). In human one-challenge intervention, human subjects were administered with LPS and at ST following LPS administration. TNF-α and IL-6 protein level in whole blood was measured. In human two-challenge intervention, human subjects were administered with LPS on 5 consecutive days and TNF-α and IL-6 protein level was measured at ST on day 5 [52]; or human subjects were administered with LPS twice with washout period of 1–2 weeks and TNF-α and IL-6 were quantified at ST following LPS administration [54, 55, 60]; or after the first *in vivo* LPS administration, whole blood extracted from the subjects was *ex vivo* treated with LPS and TNF-α and IL-6 were measured at 24h (LT) following *ex vivo* treatment [45, 51]. In mouse *in vivo* models, mice underwent CLP or sham procedure; at 24h (LT) [36] or 72h (LT) [39] after treatment macrophages or monocytes were extracted and *ex vivo* treated with LPS. At 16h (LT) [36] or 24h (LT) [39] post-*ex vivo* treatment outcomes were measured. In mouse one-challenge intervention, TNF-α protein level was measured in cells extracted from sham-treated mice, and *ex vivo* treated with LPS. In mouse two-challenge intervention, TNF-α protein expression was measured in cells extracted from CLP-treated mice, and *ex vivo* treated with LPS. Alternatively, mice were pre-treated with LPS [64] or lipoteichoic acid [61]; at 24h (LT) after first LPS or lipoteichoic acid challenge mice were treated again with LPS, and at 3h (ST) [64], 24h (LT) [61] serum or plasma TNF-α protein levels were measured for two-challenge intervention group. In one-challenge intervention group, mice were pre-treated with phosphate buffered saline for 24h, and then treated with LPS for 3h [64] or 24h [43] when outcomes were measured.

Independent meta-analyses of TNF-α and IL-6 protein expression were performed for human or rodent studies (Fig 6). Meta-analyses revealed that TNF-α and IL-6 protein levels significantly decreased in humans following two-challenges with the first *in vivo* administration with LPS and the second *ex vivo* or *in vivo* challenge with LPS compared to *in vivo* one-challenge (meta-analysis of TNF-α: six studies, P < 0.0001, I^2^ = 0%; meta-analysis of IL-6: five studies, P < 0.0001, I^2^ = 45%, Fig 6). Corresponding to the results of meta-analyses, three single studies not included due to presenting data in different formats, reported significantly lower levels of TNF-α and IL-6 protein in humans after two-challenges with LPS administration than one-challenge [53, 58, 59].

Meta-analysis of mice *in vivo* studies demonstrates significant decrease of TNF-α protein levels after two-challenges involving the first *in vivo* challenge and the second *ex vivo* or *in vivo* treatment with LPS compared to one-challenge (four studies, P = 0.02, I^2^ = 78%, Fig 6). Two mice *in vivo* studies not included in the meta-analysis due to differing challenge agents showed the same trend of down-regulated TNF-α and IL-6 protein levels following two-challenges [62, 63]. Thus, these independent meta-analyses of human or mice *in vivo* studies indicate a negative correlation between IRAK3 and TNF-α expression following two-challenge intervention (Figs 5 and 6).

#### Effect of IRAK3 on inflammatory cytokines following two-challenge intervention in mice *in vivo* studies

**Fig 7:**
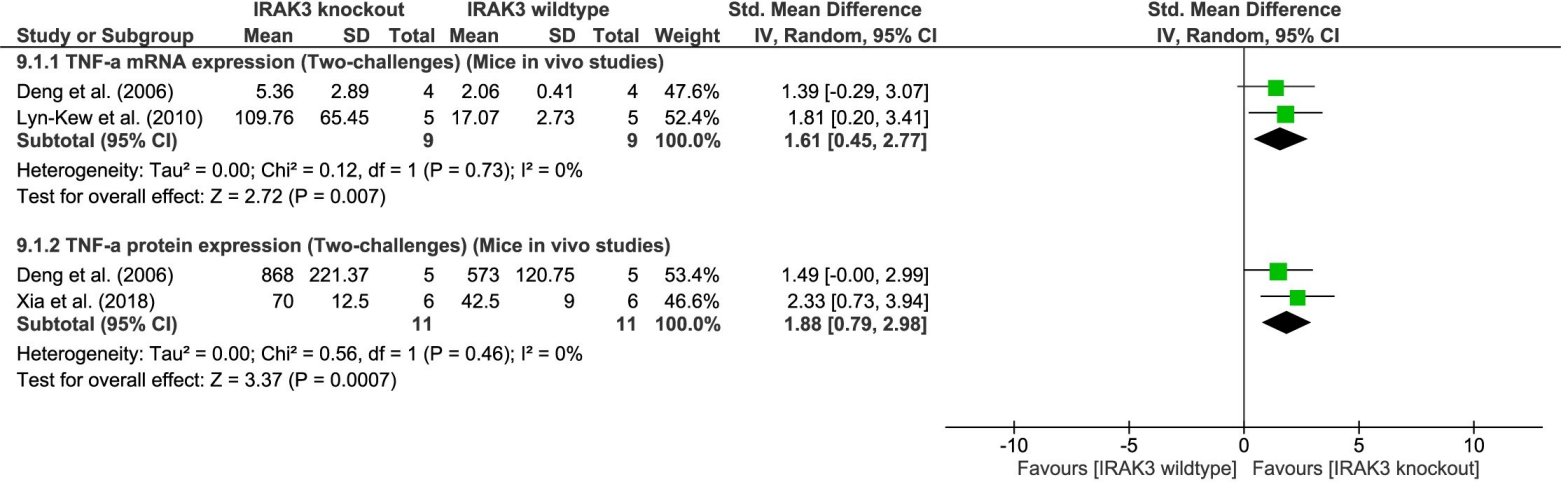
TNF-α mRNA and protein expression outcome after two-challenge intervention: IRAK3 knockout group versus IRAK3 wildtype group. Mice underwent CLP; at 24h (ST) after CLP mice were inoculated with *P*. *aeruginosa*, and at 6h (IT) after second challenge TNF-α mRNA expression was measured, and at 24h (LT) for protein expression measured [36]. Alternatively, mice underwent CLP; at 24h (LT) after CLP macrophages were extracted and *ex vivo* challenged with LPS, and at 4h (IT) after second challenge TNF-α mRNA expression was measured [38]. Similarly, mice underwent CLP; at 72h (LT) after CLP mice were injected with LPS, and at 24h (LT) after second challenge TNF-α protein expression was measured [39].

Meta-analyses of two-challenge intervention identified an inhibitory effect of IRAK3 on TNF-α mRNA and protein expression in mice (TNF-α mRNA expression: two studies, P = 0.007, I^2^ = 0%; TNF-α protein expression: two studies, P = 0.0007, I^2^ = 0%, Fig 7). IRAK3 had negative impact on TNF-α mRNA expression at 4h - 6h (IT) and 24h (LT), and on TNF-α protein level at 6h (IT) and 24h (LT) following the second challenge in mice [36] (Fig 7). There were insufficient studies using similar challenge agents to carry out meta-analysis for the effect of IRAK3 on IL-6 expression. However, an inhibitory effect of IRAK3 on IL-6 protein level in mice challenged first with CLP and then with LPS injection was observed [39]. IRAK3 was also reported to inhibit TNF-α and IL-6 protein levels in mice challenged twice, first by being exposed to cigarette smoke and then inhaling LPS [37].

## Discussion

This review identified significant increases of IRAK3 mRNA expression at IT and LT, and inflammatory cytokine (TNF-α and IL-6) levels at ST following *in vivo* challenges in both humans and rodents, and their differences between humans (e.g., IRAK3 mRNA peak levels at IT) and rodents (e.g., IRAK3 mRNA peak levels at LT). Moreover, based on meta-analyses, the review compared the findings of IRAK3 effects and cytokine expression patterns between challenged *in vitro* cell culture and *in vivo* animal models for the study of sepsis, thereby showing the translatability of these models to human. The meta-analyses of *in vivo* studies further confirmed the roles of IRAK3 during immunosuppression phase of sepsis.

Sepsis is comprised of two phases, hyper-inflammatory phase featured by dramatic elevation of potent cytokines such as TNF-α and IL-6, and immunosuppression phase where inflammatory cytokines decrease significantly [4]. IL-6 and TNF-α are inflammatory cytokines synthesized by many cell types such as monocytes, macrophages, and fibroblasts in response to infections, and play pivotal roles as signal molecules involved in various immune responses [6, 15]. Levels of IL-6 and TNF-α have been widely assessed in clinical samples under various sepsis conditions and were correlated to disease severity [17–19, 89]. Clinical samples of patients with sepsis can have highly elevated levels of TNF-α and IL-6 within one day of diagnosis [18, 19]. This phenomenon corresponds to the significant increase of TNF-α and IL-6 at ST and IT periods following LPS administration in human *in vivo* studies (Fig 3). *Ex vivo* LPS treatment of whole blood from patients diagnosed with sepsis resulted in 10–20% decreases of TNF-α and IL-6 production compared to blood from patients without sepsis [90]. The second *in vivo* or *ex vivo* administration of LPS in humans also showed lower level of circulating TNF-α and IL-6 compared to the first *in vivo* administration with LPS (Fig 6). These human *in vivo* studies correlate to general clinical findings, and effectively demonstrated the context of immunosuppression of sepsis and its underlying mechanism, endotoxin tolerance. Only two human *in vivo* studies reported IRAK3 mRNA and protein expression following one-challenge [45, 56]. IRAK3 gene expression was upregulated in blood samples from patients with sepsis, and associated with susceptibility to severe sepsis, poor outcomes and mortality [20, 91]. IRAK3 mRNA levels in survivors of sepsis were significantly lower than in non-survivors [20, 92], increased within the first five days after diagnosis, and decreased at day 28 when patients recovered [92]. Thus, the increases of IRAK3 gene expression in patients with sepsis is more extended compared to human *in vivo* models where IRAK3 mRNA expression increased at ST—IT and declined to baseline at 24h (Fig 2). Endotoxin (LPS) must be administered at non-toxic safe dose to human *in vivo* models; therefore its use is limited, and this experimental handicap can be partially overcome by using *in vivo* animal models. At ST (1h) following *ex vivo* challenge with LPS, IRAK3 mRNA expression in samples of patients with sepsis further increases compared to unchallenged samples and healthy subjects, and this corresponds to two-challenge animal *in vivo* models (Fig 5) [35]. It is necessary for more studies of IRAK3 mRNA and protein expression in human *in vivo* models, especially two-challenge intervention to be conducted to provide greater understanding of the role of IRAK3 in human sepsis and enhance treatment options with improved patient outcomes.

Many studies using *in vivo* animal (mainly rodent) models investigated IRAK3 and cytokine expression under various disease conditions (e.g., sepsis, colitis, asthma, ischemia reperfusion injury) [32, 68, 93, 94]. In this review we analysed the correlation and difference between the findings obtained from human and animal models. IRAK3 mRNA expression in humans reached peak levels at IT following one-challenge, and this is earlier than in mice at LT (Fig 2) [38, 45]. Patterns of TNF-α and IL-6 expression in human *in vivo* models are more similar to mice *in vivo* models than *in vitro* cell culture (Figs 3 and S1 and S2) [46]. IL-6 and TNF-α levels of mice at ST and IT after *in vivo* challenge were generally comparable to serum and plasma levels of humans with sepsis or septic shock (S1 and S2 Figs) [18, 19]. However, the variances of IL-6 levels in mouse models were larger than in human *in vivo* models (S2 Fig). Meta-analysis of *in vivo* IRAK3 knockout mouse model(s) showed no effect of IRAK3 on TNF-α or IL-6 protein level at ST and LT after one-challenge with LPS or bacteria (Fig 4). This is in contrast to the results of meta-analyses of data from *in vitro* cell culture studies of sepsis, which revealed inhibitory effects of IRAK3 on TNF-α level at IT in cell lines and at LT in mouse primary cells after one-challenge [46]. Both this review (Figs 5 and 6) and a prior review of *in vitro* cell studies [46] reported a negative correlation between expression of IRAK3 and protein level of inflammatory cytokines (IL-6, TNF-α) following two-challenges, which demonstrates IRAK3 contribution to the regulation of endotoxin tolerance to maintain homeostasis of inflammatory responses. The role of IRAK3 in the immunosuppressive phase is further confirmed in the meta-analyses reporting the inhibitory effect of IRAK3 on TNF-α mRNA and protein expression in mice *in vivo* and cell *in vitro* models upon two-challenges (Fig 7) [46]. Although the *in vivo* rodent models may be a more suitable indicator for the pattern of TNF-α and IL-6 protein expression of *in vivo* human models and clinical samples than *in vitro* cell models, IRAK3 knockout or silencing cell culture models showed more stable IRAK3 effects on cytokine levels than IRAK3 knockout mouse *in vivo* models [31, 40, 41, 61, 95, 96]. The contradictory findings of IRAK3 effect in *in vivo* mouse models challenged once might be caused by differences in experimental procedures, including modes of administration (e.g., inoculation with or inhalation of bacteria, LPS, CLP), bacterial species, or type and concentration of immunomodulatory agents. Notably, only one *in vivo* mouse study [32] reported inhibitory effect of IRAK3 on cytokine production after one-challenge, and used 100 to 1000-fold higher concentration of bacteria compared to other included studies [37, 40, 41]. Thus, IRAK3 may have opposing effects on cytokine levels depending on the degree of induction of immune response due to the type, dose and administration method of the challenge agent. In addition, most *in vivo* IRAK3 knockout mice studies [9, 32, 36, 37, 40, 41] had exons 9–11, encoding the pseudokinase domain, deleted. It is possible that an active truncated version of IRAK3 containing the death domain is present in these knockout mice as an IRAK3 splice variant ligating exon 8 and exon 12 has been detected [68, 97]. Notably, the pseudokinase domain also contains a cryptic guanylate cyclase centre that cell studies have shown to be involved in suppressing NFκB activity [14].

Several critical molecules associated with sepsis diagnosis or therapeutics, including triggering receptor expressed on myeloid cells-1 (TREM-1), high-mobility group box 1 protein (HMGB1), hypoxia inducible factor-1α (HIF-1α), and glucocorticoids, were reported to regulate IRAK3 expression in *in vivo* or *in vitro* models [32, 64, 98–100]. TREM-1, a surface receptor expressed on neutrophils, monocytes, and macrophages, is activated simultaneously with pattern recognition receptors such as TLRs to enhance inflammatory responses to infections [98]. HMBG1 has intracellular and extracellular roles in immune modulation activating TLR signalling [64]. Pre-treatment with TREM-1 or HMGB1 in mice stimulated with LPS or bacteria raised IRAK3 expression levels, thereby down-regulating inflammatory signalling [64, 98]. The transcription factor, HIF-1α is induced in monocytes from patients with sepsis, and upregulated IRAK3 mRNA and protein expression between 0.5h to 24h as well as reducing TNF-α and IL-6 expression at IT and LT in *in vitro* monocytes challenged with endotoxin [100]. The glucocorticoid receptor is another transcription factor that can induce IRAK3 expression, thus enhancement of IRAK3 expression is likely part of the mechanism by which glucocorticoids reduce systemic inflammatory responses [32]. The suppressive effect of glucocorticoids on inflammatory cytokines and survival rate is abolished in IRAK3 knockout mice infected with bacteria compared to wild type mice [32]. Glucocorticoids are important medicines for adjunct therapy in patients with sepsis or sepsis shock, and recently shown to be effective in treatment of severe acute respiratory syndrome associated coronavirus 2 (SARS-CoV-2) [32, 101, 102]. Additionally, expression of IRAK3 is positively correlated with gene expression of angiotensin-converting enzyme 2 receptor which is a receptor for SARS-CoV-2 entry in nasal epithelial cells [103]. Whereas SAR-CoV-2 enhanced proinflammatory cytokine levels via suppressing IRAK3 expression in macrophages and monocytes, the *in vitro* infection with Middle East respiratory syndrome coronavirus (MERS-CoV) glycoprotein suppressed TNF-α and IL-6 protein production in macrophages through mediating induction of IRAK3 expression [104, 105]. The actual expression and effect of IRAK3 in the mechanism of pathogenesis of different viral species or interspecies should be investigated in animal *in vivo* models to support the understanding of disease and immune responses caused by these viruses.

The TLR/IL1-R inflammatory cascade is also modulated by other negative regulators such as Flightless II (FLII), NOD-like receptor family, pyrin domain containing 12 (NLRP12), CD44, Toll-interacting protein (Tollip) and TRAF4. NLRP12 is a cytosolic regulator that inhibits phosphorylation of IRAK1 to decrease NF-κB signalling pathway [97], whereas TRAF4 suppresses NF-κB activity via its functional interaction with TRAF6 [106]. FLII is an actin-remodelling protein which binds to the TIR region of TLRs to inhibit the binding of MyD88 to TLRs, thus suppressing the formation of myddosome complex [107]. Tollip, an inhibitory adaptor protein associated with TLRs, down-regulates IRAK1 activity [108]. CD44 is a transmembrane adhesion molecule that negatively regulates production of inflammatory cytokines via enhancing expression of other negative regulators including IRAK3 and Tollip in macrophages [109]. Hence, IRAK3 acts cooperatively with other checkpoint molecules in TLRs/IL-1R pathway to control inflammatory responses to pathogen infections during sepsis. Further research using both human and mouse *in vivo* models on the actions and relations of IRAK3 to other molecules (glucocorticoids, TREM-1, HMBG1, and HIF-1α) and inflammatory regulators will clarify insights and may improve treatment of sepsis.

Translatability of animal studies to humans depends on external validity, that is the extent to which research findings derived in one setting, population or species can be reliably applied to other settings, populations and species [110]. For instance, both human and mouse immune responses are modified by bystander infections and shaped by pathogen-driven selection through lifetime. Thus, laboratory mice raised in specific pathogen free environment which failed to extrapolate adult human immune responses can be improved by manipulating exposure to reasonable sets of pathogens [111]. However, it is impossible to overcome insurmountable problems of external validity, and species differences in underlying biology between humans and animals lead to failures in assessing efficacy and safety of interventions at human clinical trials [110]. Though mice are the most frequently used animal models due to many biochemical and physiological similarities and high genetic homology to human, mouse models show low correlation of gene expression changes in endotoxemia to human orthologs, and poorly mimic the genomic responses in patients with sepsis and infections. A number of basic dissimilarities in immune responses between human and murine species exist [112]. For example, in humans LPS doses of 2–4 ng/kg can induce fever and cytokine production, and 15 μg/kg dose causes severe disease, but mice can develop tolerance against several TLR agonists (e.g., LPS) and require up to 10 mg/kg body mass to trigger comparable symptoms to humans [112]. Therefore, it is necessary to generate models that not only demonstrate phenotypes of specific pathology, but also the molecular basis of crucial genes, pathways, or the genome-wide level for the relevant human diseases [111, 113]. *In vitro* models of disease-related cell types or tissues should be performed to confirm IRAK3 functions in immune responses from animal studies.

Limitations of this systematic review include the high heterogeneity across studies, especially the meta-analyses of IL-6 and TNF-α protein levels following one-challenge. Nevertheless, the protein levels of IL-6 and TNF-α reported in included human *in vivo* studies were still in the value ranges of clinical samples of patients with sepsis or septic shock (Fig 3) [18, 19]. The high heterogeneity between animal studies may be explained by different models to induce illness or injury and the varying regimens of stimulant dosing with uncertain similarity to human conditions, that has been identified as a major limitation of animal research methodologies and reasons of publication bias. In addition, problems of internal validity of animal studies, including poor study design, conduct, analysis or reporting contribute to untranslatable replications in human trials [110, 114]. For instance, lack of blinding of treatments in animal studies results in overestimates of the effect of the intervention [110]. In general, most of the *in vivo* human and animal studies included in this review clearly described the procedures of experimental interventions and controls (or sham) and reported the outcome measures of intervention and control groups (S3 Table). However, most studies did not mention whether random allocation or blinded intervention and assessment were performed (S3 Table), and that can influence the validity of the studies. Another limitation of this systematic review is that several meta-analyses include only two studies due to the shortage of studies which used similar methods for investigating sepsis.

## Conclusions

**Fig 8:**
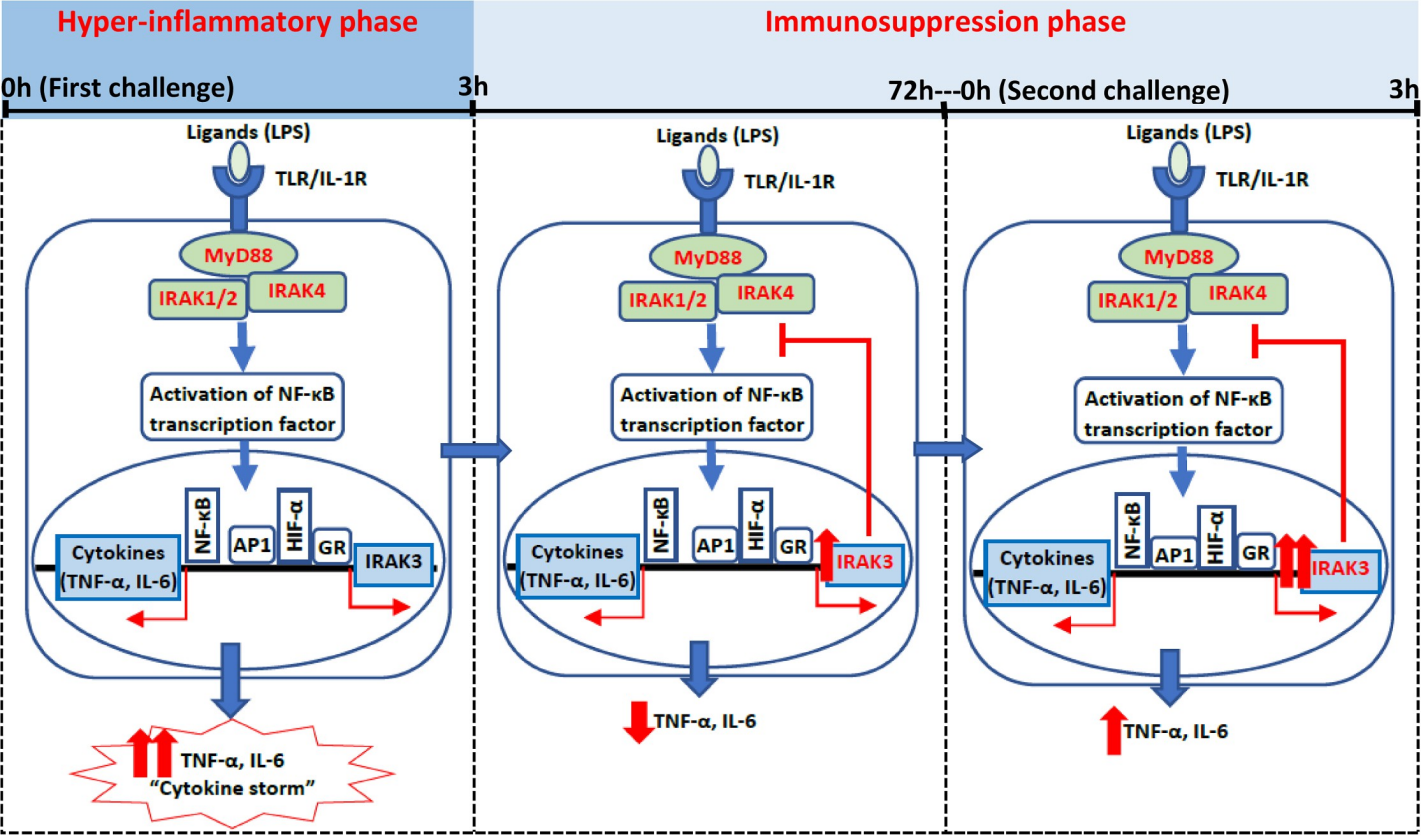
Scheme of IRAK3 and cytokine expression in humans following the first and second *in vivo* challenge. During ST (1h – 3h) after the first stimulation of TLR/IL-1R with LPS, NF-κB transcription factor is activated to induce the production of cytokines (TNF-α and IL-6), which leads to “cytokine storm”. IRAK3 expression is induced by transcription factors including activator protein-1 (AP1), hypoxia-inducible factor-α (HIF-α), glucocorticoid receptor (GR); and the peak increase of IRAK3 expression is observed at IT (4h – 15h) following the first challenge to suppress cytokine release for maintaining homeostasis. After the second challenge in human, IRAK3 expression is further increased to limit cytokine production, thus the extent of the cytokine increase after second challenge is significantly lower than the increase level after the first challenge.

This systematic review indicates a difference in the period of peak level of IRAK3 mRNA expression between human (at IT) and rodent (at LT) models. Meta-analyses showed similarities in TNF-α and IL-6 protein expression patterns between human and rodent *in vivo* studies with peak levels at ST (1h to 3h), but the levels of these cytokines in humans dropped to baseline more rapidly than in rodents. Moreover, meta-analyses of *in vivo* mouse studies following one-challenge showed no effect of IRAK3 on TNF-α and IL-6 levels, whereas IRAK3 was shown to have subtle effects based on meta-analyses of *in vitro* cell studies [46]. The inhibitory effect of IRAK3 following two-challenges was confirmed with meta-analyses by both *in vivo* and *in vitro* studies, and correlates to clinical outcomes, indicating the importance of IRAK3 in immunosuppression phase of sepsis. The expression of IRAK3 and cytokines (TNF-α and IL-6) during hyper-inflammatory and immunosuppression phases in humans is summarised in Fig 8. There is a shortage of human *in vivo* studies investigating how IRAK3 expression influences immune outcomes, especially for two-challenge intervention.

## Supporting information

S1 Fig TNF-α protein expression outcome in mice or rats: One-challenge intervention group versus control group. In one-challenge intervention, mice or rats were injected with or inhaled LPS [37, 61, 62, 66, 70, 72–77, 80, 85], or were intranasally inoculated with S. pneumoniae [41], or intraperitoneally inoculated with *E*.*coli* [79], or underwent CLP [82]. TNF-α protein expression was measured at short term (ST; 1h – 3h), intermediate term (IT; 4h – 15h), or at long term (LT; 16h – 72h).
(PDF)

S2 Fig IL-6 protein expression outcome in mice or rats: One-challenge intervention group versus control group. In one-challenge intervention, mice or rats were injected with or inhaled LPS [37, 61, 62, 66, 69, 70, 72–75, 80, 85], or were inoculated intranasally with *S*. *pneumoniae* [41], or intraperitoneally inoculated with *E*.*coli* [79], or underwent CLP [82]. IL-6 protein expression was measured at short term (ST; 1h – 3h), intermediate term (IT; 4h – 15h), or at long term (LT; 16h – 72h).
(PDF)

S1 Table Characteristics of included studies. (PDF)

S2 Table List of excluded studies and reason. (PDF)

S3 Table Summary of risk of bias in the included animal *in vivo* studies. (PDF)

S1 Checklist (DOC)
