# Supplementary figures and images for: A systematic review and meta-analyses of interleukin-1 receptor associated kinase 3 (IRAK3) action on inflammation in in vivo models for the study of sepsis
Source: PLoS One. 2022 Feb 15;17(2):e0263968. doi: 10.1371/journal.pone.0263968 (PMC8846508; doi:10.1371/journal.pone.0263968)

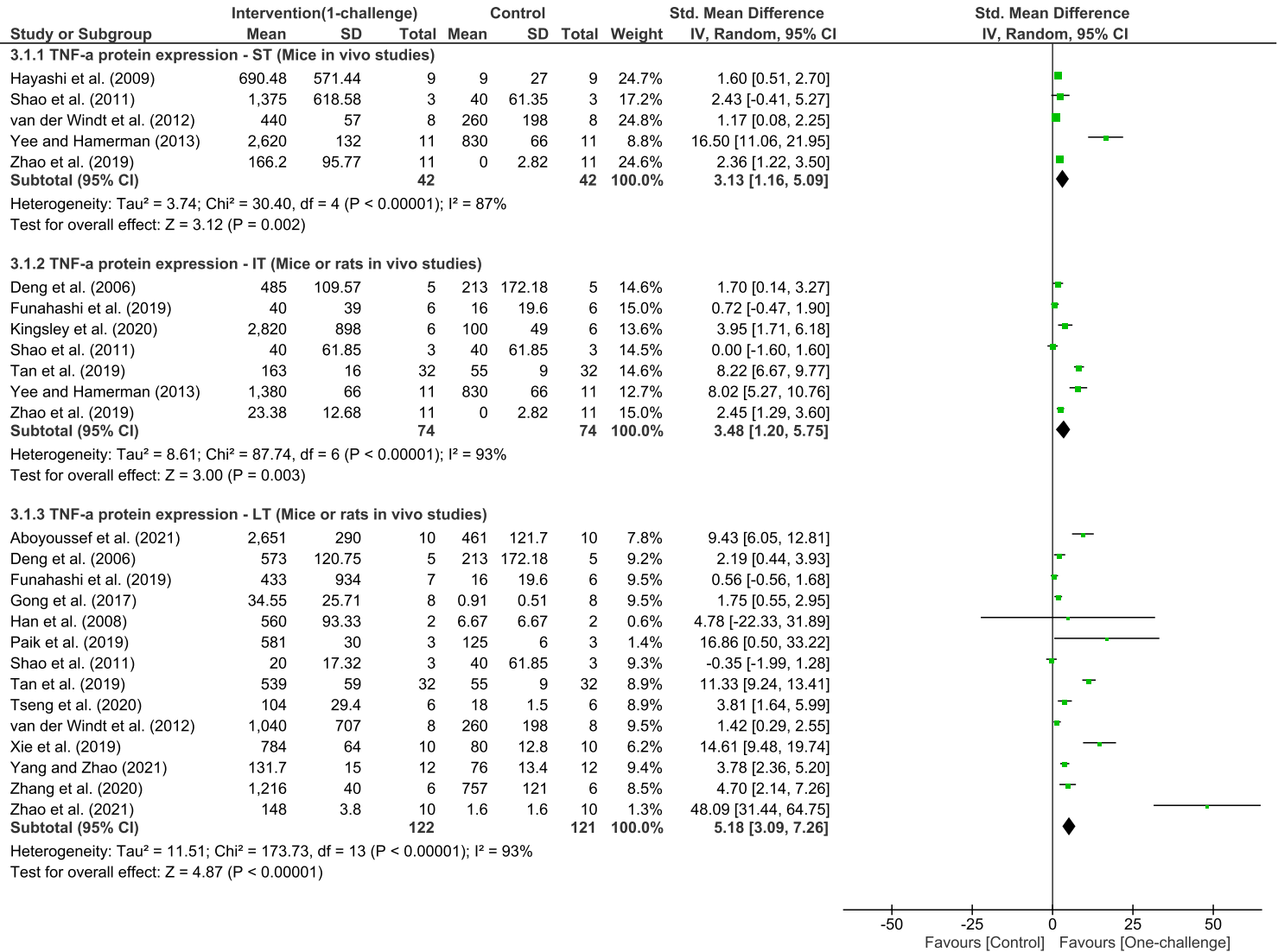

Supplement: S1 Fig — In one-challenge intervention, mice or rats were injected with or inhaled LPS [37, 61, 62, 66, 70, 72–77, 80, 85], or were intranasally inoculated with S. pneumoniae [41], or intraperitoneally inoculated with E.coli [79], or underwent CLP [82]. TNF-α protein expression was measured at short term (ST; 1h – 3h), intermediate term (IT; 4h – 15h), or at long term (LT; 16h – 72h). (PDF) [file pone.0263968.s001.pdf]

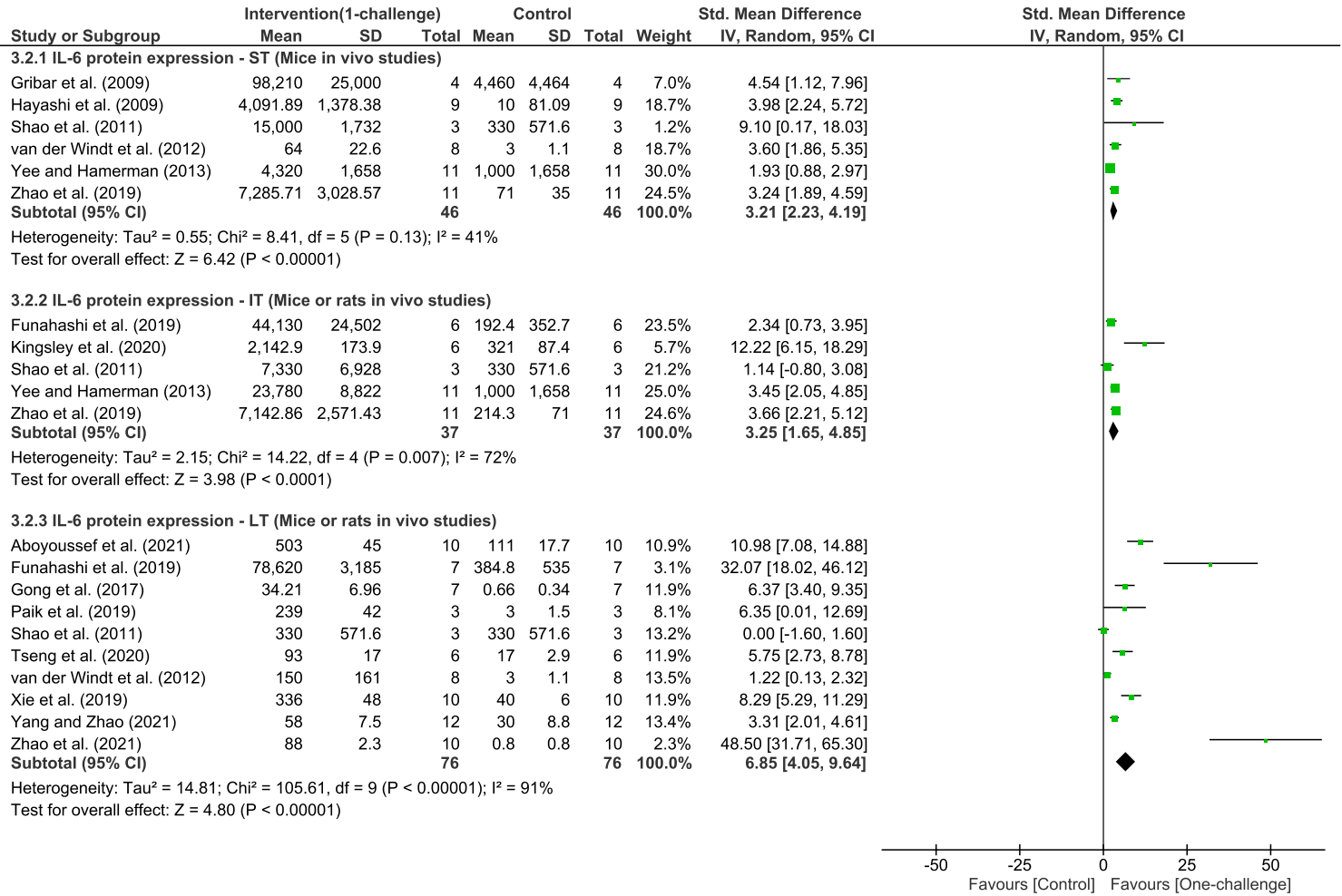

Supplement: S2 Fig — In one-challenge intervention, mice or rats were injected with or inhaled LPS [37, 61, 62, 66, 69, 70, 72–75, 80, 85], or were inoculated intranasally with S. pneumoniae [41], or intraperitoneally inoculated with E.coli [79], or underwent CLP [82]. IL-6 protein expression was measured at short term (ST; 1h – 3h), intermediate term (IT; 4h – 15h), or at long term (LT; 16h – 72h). (PDF) [file pone.0263968.s002.pdf]
