# Supplementary material for: A systematic review and meta-analyses of interleukin-1 receptor associated kinase 3 (IRAK3) action on inflammation in in vivo models for the study of sepsis
Source: PLoS One. 2022 Feb 15;17(2):e0263968. doi: 10.1371/journal.pone.0263968 (PMC8846508; doi:10.1371/journal.pone.0263968)
Supplement: S1 Table — (PDF) [file pone.0263968.s003.pdf]

S1 Table. Characteristics of included studies.

No	Study	Sample size	Treatment	Intervention duration	Comparison groups	Outcome measure of interest	Effect sizes
**Human *in vivo* studies**							
1	de Kruif *et al*. (2007)	Human healthy male subjects (n = 8)	Administration with LPS (4 ng/kg)	1.5h	Group 1: One-challenge<br>Group 2: Control	TNF-α protein expression	(Positive favours one-challenge)<br>TNF-α protein expression: 1.42 (0.29, 2.95)
				4h	Group 1: One-challenge<br>Group 2: Control	TNF-α protein expression	TNF-α protein expression: 0.00 (-0.98, 0.98)
				24h	Group 1: One-challenge<br>Group 2: Control	TNF-α protein expression	TNF-α protein expression: 0.00 (-0.98, 0.98)
				3h	Group 1: One-challenge<br>Group 2: Control	IL-6 protein expression	(Positive favours one-challenge)<br>IL-6 protein expression: 1.21 (0.11, 2.30)
				4h	Group 1: One-challenge<br>Group 2: Control	IL-6 protein expression	(Positive favours one-challenge)<br>IL-6 protein expression: 1.25 (0.15, 2.35)
2	De Vox *et al*. (2009)	Human healthy male subjects (n = 6)	Administration with LPS (4 ng/kg)	1.5h	Group 1: One-challenge<br>Group 2: Control	TNF-α protein expression	(Positive favours one-challenge)<br>TNF-α protein expression: 1.51 (0.16, 2.86)
				4h	Group 1: One-challenge<br>Group 2: Control	TNF-α protein expression	TNF-α protein expression: 0.00 (-1.13, 1.13)
				1h-2h (1st), 24h (2nd)	Group 1: Two-challenges<br>Group 2: One-challenge	TNF-α protein expression	(Negative favours one-challenge)<br>TNF-α protein expression: -0.25 (-1.38, 0.89)
						IL-6 protein expression	(Negative favours one-challenge)<br>IL-6 protein expression: -0.35 (-1.50, 0.79)
3	Draisma *et al*. (2009)	Human healthy male subjects (n = 14)	Administration with LPS (2 ng/kg)	1.5h	Group 1: One-challenge<br>Group 2: Control	TNF-α protein expression	(Positive favours one-challenge)<br>TNF-α protein expression: 4.01 (2.65, 5.36)
				4h	Group 1: One-challenge<br>Group 2: Control	TNF-α protein expression	(Positive favours one-challenge)<br>TNF-α protein expression: 0.00 (-0.74, 0.74)
				1.5h	Group 1: One-challenge<br>Group 2: Control	IL-6 protein expression	(Positive favours one-challenge)<br>IL-6 protein expression: 2.97 (1.85, 4.09)

				4h<br><br><br>1h (1st), 2h (2nd)	Group 1: One-challenge<br>Group 2: Control<br><br>Group 1: Two-challenges<br>Group 2: One-challenge	IL-6 protein expression<br><br><br>TNF-α protein expression<br><br><br>IL-6 protein expression	(Positive favours one-challenge)<br>IL-6 protein expression: 0.88 (0.10, 1.66)<br>(Negative favours one-challenge)<br>TNF-α protein expression: -0.91 (-1.69, -0.12)<br>(Negative favours one-challenge)<br>IL-6 protein expression: -2.29 (-3.28, -1.31)
4	Grondman *et al.* (2019)	Human healthy male subjects<br>(n = 15)	Administration with LPS (2 ng/kg) (1st); ex vivo monocytes stimulated with LPS (10 ng/ml) (2nd)	4h or 7d (1st); 24h (2nd)	Group 1: Two-challenges<br>Group 2: One-challenge	TNF-α protein expression<br><br>IL-6 protein expression	Not estimable<br><br>Not estimable
5	Hoogerwerf *et al.* (2008)	Human healthy male subjects<br>(n = 8)	Bronchial instillation of LPS (4 ng/kg)	6h	Group 1: One-challenge<br>Group 2: Control	IRAK3 mRNA expression	(Positive favours one-challenge)<br>IRAK3 mRNA expression: 0.70 (-0.32, 1.72)
6	Koch *et al.* (2017)	Human healthy male subjects<br>(n = 15)	Administration with LPS (2 ng/kg)	0, 1, 1.5, 2, 3, 4, 5, 6, 7, 8h	Group 1: One-challenge<br>Group 2: Control	TNF-α protein expression<br>IL-6 protein expression	Not estimable
7	Kox *et al.* (2011)	Human healthy male subjects<br>(n = 12)	Administration with LPS (4 ng/kg)	1.5h<br><br><br>4h<br><br><br>1.5h<br><br><br>4h<br><br><br>1.5h (1st), 1.5h (2nd)	Group 1: One-challenge<br>Group 2: Control<br><br>Group 1: One-challenge<br>Group 2: Control<br><br>Group 1: One-challenge<br>Group 2: Control<br><br>Group 1: One-challenge<br>Group 2: Control<br><br>Group 1: Two-challenges	TNF-α protein expression<br><br><br>TNF-α protein expression<br><br><br>IL-6 protein expression<br><br><br>IL-6 protein expression<br><br><br>TNF-α protein expression	(Positive favours one-challenge)<br>TNF-α protein expression: 1.46 (0.54, 2.38)<br>(Positive favours one-challenge)<br>TNF-α protein expression: 2.79 (1.62, 3.97)<br>(Positive favours one-challenge)<br>IL-6 protein expression: 2.06 (1.03, 3.08)<br>(Positive favours one-challenge)<br>IL-6 protein expression: 0.73 (-0.10, 1.56)<br>(Negative favours one-challenge)

			Administration with LPS (4 ng/kg)		Group 2: One-challenge	IL-6 protein expression	TNF-α protein expression: -0.94 (-1.79, -0.09)<br>(Negative favours one-challenge)<br>IL-6 protein expression: -1.18 (-2.06, -0.30)
8	Lankelma *et al*. (2017)	Human healthy male subjects<br>(n = 8)	Administration with LPS (4 ng/kg)	1.5h<br><br>4h<br><br>2h<br><br>4h	Group 1: One-challenge<br>Group 2: Control<br>Group 1: One-challenge<br>Group 2: Control<br>Group 1: One-challenge<br>Group 2: Control<br>Group 1: One-challenge<br>Group 2: Control	TNF-α protein expression<br><br>TNF-α protein expression<br><br>IL-6 protein expression<br><br>IL-6 protein expression	(Positive favours one-challenge)<br>TNF-α protein expression: 0.70 (-0.32, 1.72)<br>TNF-α protein expression: 0.00 (-0.98, 0.98)<br><br>(Positive favours one-challenge)<br>IL-6 protein expression: 0.90 (-0.14, 1.95)<br>(Positive favours one-challenge)<br>IL-6 protein expression: 0.77 (-0.25, 1.80)
9	Leentjens *et al*. (2012)	Human healthy male subjects<br>(n = 6)	Administration with LPS (2 ng/kg)	0, 2, 4, 6, 8, 24h<br>0, 2, 4, 6, 8, 24h (1st); 0, 2, 4, 6, 8, 24h (2nd)	Group 1: One-challenge<br>Group 2: Control<br>Group 1: Two-challenges<br>Group 2: One-challenge	TNF-α protein expression<br>IL-6 protein expression<br>TNF-α protein expression<br>IL-6 protein expression	Not estimable<br><br>Not estimable
10	Leijte *et al*. (2018)	Human healthy male subjects<br>(n = 10)	Administration with LPS	2.5h<br><br>4h<br><br>2.5h<br><br>4h<br><br>2.5h (1st), 3h (2nd)	Group 1: One-challenge<br>Group 2: Control<br>Group 1: One-challenge<br>Group 2: Control<br>Group 1: One-challenge<br>Group 2: Control<br>Group 1: One-challenge<br>Group 2: Control<br>Group 1: Two-challenges<br>Group 2: One-challenge	TNF-α protein expression<br><br>TNF-α protein expression<br><br>IL-6 protein expression<br><br>IL-6 protein expression<br><br>TNF-α protein expression<br><br>IL-6 protein expression	(Positive favours one-challenge)<br>TNF-α protein expression: 5.30 (3.27, 7.33)<br>(Positive favours one-challenge)<br>TNF-α protein expression: 2.96 (1.62, 4.31)<br>(Positive favours one-challenge)<br>IL-6 protein expression: 1.86 (0.83, 2.90)<br>(Positive favours one-challenge)<br>IL-6 protein expression: 1.40 (0.40, 2.41)<br>(Negative favours one-challenge)<br>TNF-α protein expression: -1.24 (-2.21, -0.26)<br>(Negative favours one-challenge)

							IL-6 protein expression: -1.51 (-2.53, -0.49)
11	Peters van Ton *et al*. (2021)	Human healthy male subjects<br>(n = 5)	Intravenous LPS challenges (2 ng/kg LPS)	1.5h	Group 1: One-challenge<br>Group 2: Control	TNF-α protein expression	(Positive favours one-challenge)<br>TNF-α protein expression: 2.33 (0.52, 4.13)
				8h	Group 1: One-challenge<br>Group 2: Control	TNF-α protein expression	(Positive favours one-challenge)<br>TNF-α protein expression: 1.22 (-0.20, 2.64)
				2h	Group 1: One-challenge<br>Group 2: Control	IL-6 protein expression	(Positive favours one-challenge)<br>IL-6 protein expression: 2.86 (0.83, 4.89)
				8h	Group 1: One-challenge<br>Group 2: Control	IL-6 protein expression	(Positive favours one-challenge)<br>IL-6 protein expression: 0.00 (-1.24, 1.24)
				1.5h, 2h (1st),<br>1.5h, 2h (2nd)	Group 1: Two-challenges<br>Group 2: One-challenge	TNF-α protein expression<br><br>IL-6 protein expression	(Negative favours one-challenge)<br>TNF-α protein expression: -1.77 (-3.36, -0.18)<br>(Negative favours one-challenge)<br>IL-6 protein expression: -2.08 (-3.78, -0.37)
12	van 't Veer *et al*. (2007)	Human healthy male subjects<br>(n = 8)	Administration with LPS (4 ng/kg)	4h	Group 1: One-challenge<br>Group 2: Control	IRAK3 mRNA expression	(Positive favours one-challenge)<br>IRAK3 mRNA expression: 1.76 (0.56, 2.97)
				1.5h	Group 1: One-challenge<br>Group 2: Control	TNF-α protein expression	(Positive favours one-challenge)<br>TNF-α protein expression: 1.75 (0.55, 2.95)
				4h	Group 1: One-challenge<br>Group 2: Control	TNF-α protein expression	TNF-α protein expression: 0.00 (-0.98, 0.98)
				24h	Group 1: One-challenge<br>Group 2: Control	TNF-α protein expression	TNF-α protein expression: 0.00 (-0.98, 0.98)
			Intravenous administration with LPS (4 ng/kg) (1st), *ex vivo* whole blood stimulated with 100 ng/mL LPS (2nd)	1-2h (1st), 24h (2nd)	Group 1: Two-challenges<br>Group 2: One-challenge	TNF-α protein expression	(Negative favours one-challenge)<br>TNF-α protein expression: -0.60 (-1.61, 0.41)
**Animal *in vivo* studies**							

1	Aboyoussef *et al.* (2021)	Adult male albino rats (n = 10)	Cecal ligation and puncture (CLP)	24h	Group 1: One-challenge<br>Group 2: Control	TNF-α protein expression<br><br>IL-6 protein expression	(Positive favours one-challenge)<br>TNF-α protein expression: 9.43 (6.05, 12.81)<br>(Positive favours one-challenge)<br>IL-6 protein expression: 10.98 (7.08, 14.88)
2	Berglund *et al.* (2010)	C57BL/6 mice (n = 5-10)	3% DSS dissolved in tap water for a period of 5d followed by 2d of regular drinking water	7d	Group 1: IRAK3 knockout<br>Group 2: IRAK3 wildtype	TNF-α and IL-6 mRNA and protein expression	Not estimable
3	Deng *et al.* (2006)	C57BL/6J mice (n = 5)	Cecal ligation and puncture (CLP)	24h	Group 1: One-challenge<br>Group 2: Control	IRAK3 mRNA expression	(Positive favours one-challenge)<br>IRAK3 mRNA expression: 15.59 (4.78, 26.40)
			Inoculation with *P. aeruginosa*	24h	Group 1: One-challenge<br>Group 2: Control	TNF-α protein expression	(Positive favours one-challenge)<br>TNF-α protein expression: 2.19 (0.44, 3.93)
				6h	Group 1: One-challenge<br>Group 2: Control	TNF-α protein expression	(Positive favours one-challenge)<br>TNF-α protein expression: 1.70 (0.14, 3.27)
			CLP (1st); lung macrophages extracted and *ex vivo* challenged with LPS (1 µg/ml) (2nd)	24h (1st), 6h (2nd)	Group 1: Two-challenges<br>Group 2: One-challenge	IRAK3 mRNA expression	(Positive favours two-challenge)<br>IRAK3 mRNA expression: 1.14 (-0.45, 2.73)
			CLP (1st), macrophages extracted and *ex vivo* challenged with LPS (100 ng/ml) (2nd)	24h (1st), 16h (2nd)	Group 1: Two-challenges<br>Group 2: One-challenge	TNF-α protein expression	(Negative favours one-challenge)<br>TNF-α protein expression: -1.05 (-2.42, 0.32)
			CLP (1st), inoculation with	24h (1st), 6h (2nd)	Group 1: IRAK3 knockout	TNF-α mRNA expression	(Positive favours IRAK3 knockout)

			P. aeruginosa (2nd)<br><br>CLP (1st), inoculation with *P. aeruginosa* (2nd)	24h (1st), 24h (2nd)	Group 2: IRAK3 wildtype<br><br>Group 1: IRAK3 knockout<br>Group 2: IRAK3 wildtype	TNF-α protein expression	TNF-α mRNA expression: 1.39 (-0.29, 3.07)<br><br>(Positive favours IRAK3 knockout)<br>TNF-α protein expression: 1.49 (0.00, 2.99)
4	Funahashi *et al*. (2019)	C57BL/6 mice (n = 6-7)	CLP	6h<br><br>24h<br><br><br><br>6h<br><br>24h	Group 1: One-challenge<br>Group 2: Control<br><br><br><br><br>Group 1: One-challenge<br>Group 2: Control	TNF-α protein expression<br><br><br><br><br><br>IL-6 protein expression	(Positive favours one-challenge)<br>TNF-α protein expression: 0.72 (-0.47, 1.90)<br>(Positive favours one-challenge)<br>TNF-α protein expression: 0.56 (-0.56, 1.68)<br>(Positive favours one-challenge)<br>IL-6 protein expression: 2.34 (0.73, 3.95)<br>(Positive favours one-challenge)<br>IL-6 protein expression: 32.07 (18.02, 46.12)
5	Gandhirajan *et al.* (2021)	C57BL/6 mice<br>(n = 5)	Intraperitoneally injected 250 µl cecal slurry (CS)-induced sepsis	4h, 24h	Group 1: One-challenge<br>Group 2: Control	TNF-α and IL-6 protein expression	Not estimable
6	Gong *et al*. (2017)	C57BL/6 mice<br>(n = 8)	Inhalation with LPS (100 µg/mL) for 30min	24h (outcomes measured after challenge)	Group 1: One-challenge<br>Group 2: Control<br><br>Group 1: One-challenge<br>Group 2: Control<br><br>Group 1: IRAK3 knockout<br>Group 2: IRAK3 wildtype	TNF-α protein expression<br><br><br>IL-6 protein expression<br><br><br>TNF-α protein expression<br><br><br><br>IL-6 protein expression	(Positive favours one-challenge)<br>TNF-α protein expression: 1.75 (0.55, 2.95)<br>(Positive favours one-challenge)<br>IL-6 protein expression: 6.37 (3.40, 9.35)<br>(Positive favours IRAK3 knockout)<br>TNF-α protein expression: 0.24 (-0.74, 1.23)<br>(Negative favours IRAK3 wildtype)

			Cigarette smoking exposure (1st), inhalation with LPS (100 µg/mL) for 30min (2nd)	3d (1st), 24h (2nd)	Group 1: IRAK3 knockout<br>Group 2: IRAK3 wildtype	TNF-α protein expression<br>IL-6 protein expression	IL-6 protein expression: -0.93 (-2.05, 0.19)<br>Not estimable.
7	Gribar *et al.* (2009)	C57BL/6 mice<br>(n = 4)	Injected with LPS (5 mg/kg)	3h	Group 1: One-challenge<br>Group 2: Control	IL-6 protein expression	(Positive favours one-challenge)<br>IL-6 protein expression: 4.54 (1.12, 7.96)
8	Han *et al.* (2008)	BALB/c mice (n = 2)	Injection with 45 mg/kg LPS	24h	Group 1: One-challenge<br>Group 2: Control	TNF-α protein expression	(Positive favours one-challenge)<br>TNF-α protein expression: 4.78 (-22.33, 31.89)
			Injection with 15 mg/kg LPS or 300 mg/kg pLTA (1st), injection with 45 mg/kg LPS (2nd)	24h (1st), 24h (2nd)	Group 1: Two-challenges<br>Group 2: One-challenge	TNF-α protein expression	(Negative favours one-challenge)<br>TNF-α protein expression: -3.43 (-22.96, 16.09)
9	Hayashi *et al.* (2009)	C57BL/6 mice<br>(n = 8-10)	Injection with LPS (2 µg per mouse)	2h	Group 1: One-challenge<br>Group 2: Control	TNF-α protein expression	(Positive favours one-challenge)<br>TNF-α protein expression: 1.60 (0.51, 2.70)
			Injection with LPS (2 µg per mouse)	2h	Group 1: One-challenge<br>Group 2: Control	IL-6 protein expression	(Positive favours one-challenge)<br>IL-6 protein expression: 3.98 (2.24, 5.72)
			Injection with 1V136, Pam3CSK4 or pIC	2-3h	Group 1: One-challenge<br>Group 2: Control	TNF-α and IL-6 protein expression	Not estimable
			Injection with 1V136 (1st), mice injected with 1V136, Pam3CSK4, pIC, LPS or ISS-ODN (2nd)	3d (1st), 2h (2nd)	Group 1: Two-challenges<br>Group 2: One-challenge	TNF-α and IL-6 protein expression	Not estimable

10	Hoogerwerf *et al*. (2012)	C57BL/6<br>(n = 8)	Infection with 1 × 10^4 CFU of *K. pneumoniae*	24h<br><br>3h<br><br>3h<br><br>24h<br><br>24h	Group 1: One-challenge<br>Group 2: Control<br><br>Group 1: IRAK3 knockout<br>Group 2: IRAK3 wildtype<br><br>Group 1: IRAK3 knockout<br>Group 2: IRAK3 wildtype<br><br>Group 1: IRAK3 knockout<br>Group 2: IRAK3 wildtype<br><br>Group 1: IRAK3 knockout<br>Group 2: IRAK3 wildtype	IRAK3 mRNA expression<br><br>TNF-α protein expression<br><br>IL-6 protein expression<br><br>TNF-α protein expression<br><br>IL-6 protein expression	(Positive favours one-challenge)<br>IRAK3 mRNA expression: 2.84 (1.34, 4.34)<br><br>(Positive favours IRAK3 knockout)<br>TNF-α protein expression: 0.46 (-0.54, 1.45)<br><br>(Positive favours IRAK3 knockout)<br>IL-6 protein expression: 0.57 (-0.43, 1.58)<br><br>(Negative favours IRAK3 wildtype)<br>TNF-α protein expression: -0.65 (-0.54, 1.45)<br><br>(Negative favours IRAK3 wildtype)<br>IL-6 protein expression: -1.68 (-2.86, -0.49)
11	Kallapur *et al*. (2007)	Sheep<br>(n = 5 – 9/ group)	Injection with LPS (10 mg) twice (1st), lung or blood monocytes extracted and in vitro stimulated with LPS (100 ng/mL) (2nd)	2d and 7d (1st)<br>16h (2nd)	Group 1: Two-challenges<br>Group 2: One-challenge	IRAK3 protein expression	Not estimable
12	Kim *et al*. (2008)	BALB/c mice<br>(n = 3)	Injection with 10 – 40 µg/mouse CpGDNA<br>Injection with 10 – 40 µg/mouse	24h<br><br>24h (1st), 6h (2nd)	Group 1: One-challenge<br>Group 2: Control<br><br>Group 1: Two-challenges<br>Group 2: One-challenge	IRAK3 protein expression<br><br>TNF-α and IL-6 protein expression	Not estimable<br><br>Not estimable

			CpGDNA (1st), CpGDNA (2nd)				
13	Kingsley *et al*. (2020)	Rats (n = 6)	Inoculation with 600 CFU of *E. coli*.	12h	Group 1: One-challenge<br>Group 2: Control	TNF-α and IL-6 protein expression	(Positive favours one-challenge)<br>TNF-α protein expression: 3.95 (1.71, 6.78)<br>(Positive favours one-challenge)<br>IL-6 protein expression: 12.22 (6.15, 18.29)
14	Krogmann *et al*. (2020)	C57BL/6J mice<br>(n = 2-4)	Injected mice with 20-100 µg R848 (TLR7 ligand)	2h	Group 1: One-challenge<br>Group 2: Control	IL-6 protein expression	Not estimable
15	Li *et al*. (2013)	BALB/c mice<br>(n = 3-6)	Injection with LPS (0.2 mg/kg) (1st), injection with LPS (10 mg/kg) (2nd)	24h (1st), 3h (2nd)	Group 1: Two-challenges<br>Group 2: One-challenge	IRAK3 protein expression (n = 3)<br><br>TNF-α protein expression (n = 6)	(Positive favours two-challenge)<br>IRAK3 protein expression: 5.71 (-0.03, 11.45)<br>(Negative favours one-challenge)<br>TNF-α protein expression: -11.99 (-17.95, -6.03)
16	Li *et al*. (2016)	Pig<br>(n = 3)	Oral administration with *E. coli* (1 × 10^9 CFU/kg).<br><br>Diet supplemented with *L. acidophilus* (1st), oral administration with *E. coli* (1 × 10^9 CFU/kg)	3h<br><br>14d (1st), 3h (2nd)	Group 1: One-challenge<br>Group 2: Control<br><br>Group 1: Two-challenges<br>Group 2: One-challenge	IRAK3 mRNA expression<br>TNF-α protein expression<br><br>IRAK3 mRNA expression<br>TNF-α protein expression	Not estimable<br><br>Not estimable
17	Lyn-Kew *et al*. (2010)	C57BL/6J mice<br>(n = 5)	CLP	24h<br><br>24h	Group 1: One-challenge<br>Group 2: Control<br><br>Group 1: IRAK3 knockout	IRAK3 mRNA expression<br><br>TNF-α mRNA expression	(Positive favours one-challenge)<br>IRAK3 mRNA expression: 4.29 (-0.15, 8.72)<br>(Positive favours IRAK3 knockout)

			CLP (1st); pulmonary macrophages extracted and stimulated 1 mg/ml LPS (n = 3)	24h (1st), 4h (2nd)	Group 2: IRAK3 wildtype<br>Group 1: Two-challenges<br>Group 2: One-challenge<br><br>Group 1: IRAK3 knockout<br>Group 2: IRAK3 wildtype	IRAK3 mRNA expression<br><br><br><br>TNF-α mRNA expression	TNF-α mRNA expression: 1.16 (-0.24, 2.57)<br>(Positive favours two-challenges)<br>IRAK3 mRNA expression: 4.06 (-0.17, 8.29)<br><br>(Positive favours IRAK3 knockout)<br>TNF-α mRNA expression: 1.81 (0.20, 3.41)
18	Miyata *et al.* (2015)	C57BL/6J mice	Inoculation with 1x10^7 to 5x10^8 CFU of *Haemophilus influenzae*	24h	Group 1: One-challenge<br>Group 2: Control<br><br><br>Group 1: IRAK3 knockout<br>Group 2: IRAK3 wildtype<br><br>Group 1: IRAK3 knockout<br>Group 2: IRAK3 wildtype<br><br>Group 1: IRAK3 knockout<br>Group 2: IRAK3 wildtype	IRAK3 mRNA expression<br><br><br><br>TNF-α mRNA expression<br><br><br>TNF-α protein expression<br><br><br>IL-6 protein expression	(Positive favours one-challenge)<br>IRAK3 mRNA expression: 9.61 (0.19, 19.03)<br><br>(Positive favours IRAK3 knockout)<br>TNF-α mRNA expression: 1.84 (-0.55, 4.23)<br>(Positive favours IRAK3 knockout)<br>TNF-α protein expression: 1.74 (0.62, 2.87)<br>(Positive favours IRAK3 knockout)<br>IL-6 protein expression: 1.73 (0.60, 2.85)
19	Paik *et al.* (2019)	C57BL/6 mice<br>(n = 3)	Administration with LPS (30 mg/kg)	18h	Group 1: One-challenge<br>Group 2: Control	TNF-α protein expression<br><br><br>IL-6 protein expression	(Positive favours one-challenge)<br>TNF-α protein expression: 16.86 (0.50, 33.22)<br>(Positive favours one-challenge)<br>IL-6 protein expression: 6.35 (0.01, 12.69)
20	Petzl *et al.* (2012)	Cow	Inoculation with *E. coli.*	24h	Group 1: One-challenge<br>Group 2: Control	IRAK3 mRNA expression	Not estimable

			Inoculation with LPS (1st), inoculation with *E. coli* (2nd).	72h or 240h (1st), 24h (2nd)	Group 1: Two-challenges<br>Group 2: One-challenge	IRAK3 mRNA expression	Not estimable
21	Rothschild *et al.* (2017)	Mice<br>(n = 3-4)	3-5% DSS dissolved in tap water for a period of 5d followed by 2d of regular drinking water	7d	Group 1: IRAK3 knockout<br>Group 2: IRAK3 wildtype	IL-6 protein expression	Not estimable
22	Shao *et al.* (2011)	C57BL/6 mice<br>(n = 3)	Injection with LPS (0.1-0.5 μg/g)	1h<br><br>6h<br><br>24h<br><br>1h<br><br>6h<br><br>24h	Group 1: One-challenge<br>Group 2: Control<br>Group 1: One-challenge<br>Group 2: Control<br>Group 1: One-challenge<br>Group 2: Control<br>Group 1: One-challenge<br>Group 2: Control<br>Group 1: One-challenge<br>Group 2: Control<br>Group 1: One-challenge<br>Group 2: Control	TNF-α protein expression<br><br>TNF-α protein expression<br><br>TNF-α protein expression<br><br>TNF-α protein expression<br><br>TNF-α protein expression<br><br>TNF-α protein expression	(Positive favours one-challenge)<br>TNF-α protein expression: 2.43 (-0.41, 5.27)<br>TNF-α protein expression: 0.00 (-0.16, 0.16)<br>(Negative favours control)<br>TNF-α protein expression: -0.35 (-1.99, 1.28)<br>(Positive favours one-challenge)<br>TNF-α protein expression: 9.10 (0.17, 18.03)<br>(Positive favours one-challenge)<br>TNF-α protein expression: 1.14 (-0.80, 3.08)<br>TNF-α protein expression: 0.00 (-1.60, 1.60)
23	Szatanik *et al.* (2011)	BALB/c mice (n = 6)	Injection with $5x10^6$ CFU of *N. meningitides*	2h, 6h, 24h	Group 1: One-challenge group<br>Group 2: lack of control	TNF-α and IL-6 protein expression	Not estimable
24	Takebayashi *et al.* (2009)	IQI/Jic mice (n = 6)	Intraperitoneal administration with 0.3 – 0.4 mg LPS	2h - 3h	Group 1: One-challenge<br>Group 2: Control	IRAK3 mRNA expression	Not estimable
25	Tan *et al.* (2019)	Wistar rats	Injection with LPS (0.4 mg/rat)	4h	Group 1: One-challenge<br>Group 2: Control	TNF-α protein expression	(Positive favours one-challenge)

				16h			TNF-α protein expression: 8.22 (6.67, 9.77)<br>(Positive favours one-challenge)<br>TNF-α protein expression: 11.33 (9.24, 13.41)
26	Tseng *et al.* (2020)	Sprague-Dawley rats (n = 6)	Injection with LPS (10 mg/kg)	24h	Group 1: One-challenge<br>Group 2: Control	TNF-α protein expression<br><br>IL-6 protein expression	(Positive favours one-challenge)<br>TNF-α protein expression: 3.81 (1.64, 5.99)<br>(Positive favours one-challenge)<br>IL-6 protein expression: 5.75 (2.73, 8.78)
27	van der Windt *et al.* (2012)	C57BL/6 mice (n = 8)	Inoculation with 5×10^4 CFUs of *S. pneumoniae*	3h<br><br>24h<br><br>3h<br><br>24h<br><br>3h<br><br>24h<br><br>3h<br><br>24h	Group 1: One-challenge<br>Group 2: Control<br>Group 1: One-challenge<br>Group 2: Control<br>Group 1: One-challenge<br>Group 2: Control<br>Group 1: One-challenge<br>Group 2: Control<br>Group 1: IRAK3 knockout<br>Group 2: IRAK3 wildtype<br>Group 1: IRAK3 knockout<br>Group 2: IRAK3 wildtype<br>Group 1: IRAK3 knockout<br>Group 2: IRAK3 wildtype	TNF-α protein expression<br><br>TNF-α protein expression<br><br>IL-6 protein expression<br><br>IL-6 protein expression<br><br>TNF-α protein expression<br><br>TNF-α protein expression<br><br>TNF-α protein expression<br><br>IL-6 protein expression	(Positive favours one-challenge)<br>TNF-α protein expression: 1.17 (0.08, 2.25)<br>(Positive favours one-challenge)<br>TNF-α protein expression: 1.42 (0.29, 2.55)<br>(Positive favours one-challenge)<br>IL-6 protein expression: 3.60 (1.86, 5.35)<br>(Positive favours one-challenge)<br>IL-6 protein expression: 1.22 (0.13, 2.32)<br>(Positive favours IRAK3 knockout)<br>TNF-α protein expression: 0.13 (-0.85, 1.11)<br>(Positive favours IRAK3 knockout)<br>TNF-α protein expression: 0.88 (-0.90, 1.06)<br>(Positive favours IRAK3 knockout)<br>IL-6 protein expression: 0.13 (-0.85, 1.11)

					Group 1: IRAK3 knockout<br>Group 2: IRAK3 wildtype	IL-6 protein expression	(Negative favours IRAK3 wildtype)<br>IL-6 protein expression: -0.09 (-1.07, 0.89)
28	Xia *et al.* (2018)	C57BL/6 mice<br>(n = 6)	CLP (1st), injection with LPS (10 mg/kg) (2nd)	3d (1st), 24h (2nd)	Group 1: Two-challenges<br>Group 2: One-challenge	IRAK3 mRNA expression<br><br>TNF-α protein expression	(Positive favours two-challenge)<br>IRAK3 mRNA expression: 4.91 (2.26, 7.56)<br>(Negative favours one-challenge)<br>TNF-α protein expression: -2.84 (-4.63, 1.05)
					Group 1: IRAK3 knockout<br>Group 2: IRAK3 wildtype	TNF-α protein expression	(Positive favours IRAK3 knockout)<br>TNF-α protein expression: 2.33 (0.73, 3.94)
29	Xie *et al.* (2019)	C57BL/6 mice<br>(n = 10)	Injection with LPS (40 μg/kg)	24h	Group 1: One-challenge<br>Group 2: Control	TNF-α protein expression<br><br>IL-6 protein expression	(Positive favours one-challenge)<br>TNF-α protein expression: 14.61 (9.48, 19.74)<br>(Positive favours one-challenge)<br>IL-6 protein expression: 8.02 (5.11, 10.92)
30	Xiong *et al.* (2011)	C57BL/6J mice<br>(n = 3)	Inoculation with LPS (25 μg/ mouse)	24h	Group 1: One-challenge<br>Group 2: Control	IRAK3 mRNA expression	(Positive favours one-challenge)<br>IRAK3 mRNA expression: 6.36 (0.01, 12.71)
			Inoculation with LPS (25 μg/ mouse), macrophages extracted and treated with 100 ng/ml LPS	24h (1st), 3 h (2nd)	Group 1: Two-challenges<br>Group 2: One-challenge	TNF-α mRNA expression	Not estimable
31	Yang and Zhao (2021)	Rats (n = 12)	Injection with LPS (3mg/kg)	48h	Group 1: One-challenge<br>Group 2: Control	TNF-α and IL-6 protein expression	(Positive favours one-challenge)<br>TNF-α protein expression: 3.78 (2.36, 5.20)<br>(Positive favours one-challenge)

							IL-6 protein expression: 3.31 (2.01, 4.61)
32	Yee and Hameman (2013)	C57BL/6 mice	Injection with LPS (1 mg/kg)	2h<br><br>4h<br><br>2h<br><br>4h	Group 1: One-challenge<br>Group 2: Control<br><br>Group 1: One-challenge<br>Group 2: Control<br><br>Group 1: One-challenge<br>Group 2: Control<br><br>Group 1: One-challenge<br>Group 2: Control	TNF-α protein expression<br><br>TNF-α protein expression<br><br>IL-6 protein expression<br><br>IL-6 protein expression	(Positive favours one-challenge)<br>TNF-α protein expression: 16.50 (11.06, 21.95)<br>(Positive favours one-challenge)<br>TNF-α protein expression: 8.02 (5.27, 10.76)<br>(Positive favours one-challenge)<br>IL-6 protein expression: 1.93 (0.88, 2.97)<br>(Positive favours one-challenge)<br>IL-6 protein expression: 3.45 (2.05, 4.85)
33	Yu *et al.* (2017)	Wistar rats	Injection with LPS (200 µg per mouse)<br><br>Daily injection with LPS (0.1 mg/kg LPS) (1st), on day 6 injection with LPS (200 µg per mouse)	24h<br><br>5d (1st), 24h (2nd).	Group 1: One-challenge<br>Group 2: Control<br><br>Group 1: Two-challenges<br>Group 2: One-challenge	IRAK3 mRNA expression<br><br>IRAK3 protein expression	(Positive favours one-challenge)<br>IRAK3 mRNA expression: 4.78 (2.91, 6.66)<br><br>(Positive favours two-challenge)<br>IRAK3 protein expression: 3.03 (1.67, 4.40)
34	Zhang *et al.* (2020)	C57BL/6 mice (n = 6)	CLP	24h	Group 1: One-challenge<br>Group 2: Control	TNF-α protein expression	(Positive favours one-challenge)<br>TNF-α protein expression: 4.28 (1.91, 6.66)
35	Zhang and Wang (2021)	Male Sprague-Dawley rats (n = 6)	5.5% (w/v) dextran sodium sulfate-treated rats	7d	Group 1: One-challenge<br>Group 2: Control	TNF-α and IL-6 protein expression	Not estimable
36	Zhao *et al.* (2019)	BALB/c mice (n = 11)	Injection with LPS (10mg/kg)	2h<br><br>6h	Group 1: One-challenge<br>Group 2: Control<br><br>Group 1: One-challenge<br>Group 2: Control	TNF-α protein expression<br><br>TNF-α protein expression	(Positive favours one-challenge)<br>TNF-α protein expression: 2.36 (1.22, 3.50)<br>(Positive favours one-challenge)

				2h<br><br>6h	Group 1: One-challenge<br>Group 2: Control<br><br>Group 1: One-challenge<br>Group 2: Control	IL-6 protein expression<br><br>IL-6 protein expression	TNF-α protein expression: 2.45 (1.29, 3.60)<br>(Positive favours one-challenge)<br>IL-6 protein expression: 3.24 (1.89, 4.59)<br>(Positive favours one-challenge)<br>IL-6 protein expression: 3.66 (2.21, 5.12)
37	Zhao *et al.* (2021)	BALB/c mice (n = 10)	Injection with LPS (2mg/kg)	24h	Group 1: One-challenge<br>Group 2: Control	TNF-α protein expression<br><br>IL-6 protein expression	(Positive favours one-challenge)<br>TNF-α protein expression: 48.09 (31.44, 64.75)<br>(Positive favours one-challenge)<br>IL-6 protein expression: 48.50 (31.71, 65.30)
