# Supplementary material for: A systematic review and meta-analyses of interleukin-1 receptor associated kinase 3 (IRAK3) action on inflammation in in vivo models for the study of sepsis
Source: PLoS One. 2022 Feb 15;17(2):e0263968. doi: 10.1371/journal.pone.0263968 (PMC8846508; doi:10.1371/journal.pone.0263968)
Supplement: S2 Table — (PDF) [file pone.0263968.s004.pdf]

S2 Table. List of excluded studies and reason.

Reasons for exclusions	Articles
Not relevant data.	1. Al-Qahtani AA, Lyroni K, Aznaourova M, Tseliou M, Al-Anazi MR, Al-Ahdal MN, *et al*. Middle east respiratory syndrome corona virus spike glycoprotein suppresses macrophage responses via DPP4-mediated induction of IRAK-M and PPARγ. *Oncotarget*. (2017) 8(6):9053-66.<br>2. Balaci L, Spada MC, Olla N, Sole G, Loddo L, Anedda F, *et al*. IRAK-M is involved in the pathogenesis of early-onset persistent asthma. *Am J Hum Genet*. (2007) 80(6):1103-14.<br>3. Dong GH, Gong JP, Li JZ, Luo YH, Li ZD, Li PZ, *et al*. Association between gene polymorphisms of IRAK-M and the susceptibility of sepsis. *Inflammation*. (2013) 36(5):1087-93.<br>4. Gribar SC, Sodhi CP, Richardson WM, Anand RJ, Gittes GK, Branca MF, *et al*. Reciprocal expression and signaling of TLR4 and TLR9 in the pathogenesis and treatment of necrotizing enterocolitis. *J Immunol*. (2009) 182(1):636-46.<br>5. Hulsmans M, Geeraert B, De Keyzer D, Mertens A, Lannoo M, Vanaudenaerde B, *et al*. Interleukin-1 receptor-associated kinase-3 is a key inhibitor of inflammation in obesity and metabolic syndrome. *PLoS One*. (2012) 7(1):e30414.<br>6. Hulsmans M, Van Dooren E, Mathieu C, Holvoet P. Decrease of miR-146b-5p in monocytes during obesity is associated with loss of the anti-inflammatory but not insulin signaling action of adiponectin. *PLoS One*. (2012) 7(2):e32794.<br>7. Jin P, Bo L, Liu Y, Lu W, Lin S, Bian J, *et al*. Activator protein 1 promotes the transcriptional activation of IRAK-M. *Biomed Pharmacother*. (2016) 83:1212-9. doi: 10.1016/j.biopha.2016.08.024.<br>8. Kim YI, Park JE, Kwon KH, Hong CY, Yi AK. Interleukin-1 receptor-associated kinase 2- and protein kinase D1-dependent regulation of IRAK-monocyte expression by CpG DNA. *PLoS One*. (2012) 7(8).<br>9. Kobayashi H, Nolan A, Naveed B, Hoshino Y, Segal LN, Fujita Y, *et al*. Neutrophils activate alveolar macrophages by producing caspase-6-mediated cleavage of IL-1 receptor-associated kinase-M. *J Immunol*. (2011) 186(1):403-10.<br>10. Learn CA, Boger MS, Li L, McCall CE. The phosphatidylinositol 3-kinase pathway selectively controls sIL-1RA not interleukin-1β production in the septic leukocytes. *J Biol Chem*. (2001) 276(23):20234-9.<br>11. Lee SA, Fitzgerald SM, Huang SK, Li C, Chi DS, Milhorn DM, *et al*. Molecular regulation of interleukin-13 and monocyte chemoattractant protein-1 expression in human mast cells by interleukin-1β. *Am J Respir Cell Mol Biol*. (2004) 31(3):283-91.<br>12. Li L, Cousart S, Hu J, McCall CE. Characterization of interleukin-1 receptor-associated kinase in normal and endotoxin-tolerant cells. *J Biol Chem*. (2000) 275(30):23340-5.<br>13. Lu X, Xue L, Sun W, Ye J, Zhu Z, Mei H. Identification of key pathogenic genes of sepsis based on the gene expression Omnibus database. *Mol Med Rep*. (2018) 17(2):3042-54.<br>14. Maitra U, Li L. Molecular mechanisms responsible for the reduced expression of cholesterol transporters from macrophages by low-dose endotoxin. *Arterioscler Thromb Vasc Biol*. (2013) 33(1):24-33.<br>15. Nanthakumar N, Meng D, Goldstein AM, Zhu W, Lu L, Uauy R, *et al*. The mechanism of excessive intestinal inflammation in necrotizing enterocolitis: An immature innate immune response. *PLos One*. (2011) 6(3): e17776.<br>16. Patenaude J, D'Elia M, Côté-Maurais G, Bernier J. LPS response and endotoxin tolerance in Flt-3L-induced bone marrow-derived dendritic cells. *Cell Immunol*. (2011) 271(1):184-91.<br>17. Pathak SK, Basu S, Bhattacharyya A, Pathak S, Kundu M, Basu J. *Mycobacterium tuberculosis* lipoarabinomannan-mediated IRAK-M induction negatively regulates Toll-like receptor-dependent interleukin-12 p40 production in macrophages. *J Biol Chem*. (2005) 280(52):42794-800.

	18. Peng Q, O'Loughlin JL, Humphrey MB. DOK3 negatively regulates LPS responses and endotoxin tolerance. *PLoS One*. (2012) 7(6):e39967. doi: 10.1371/journal.pone.0039967.<br>19. Pino-Yanes M, Ma SF, Sun X, Tejera P, Corrales A, Blanco J, *et al*. Interleukin-1 receptor-associated kinase 3 gene associates with susceptibility to acute lung injury. *Am J Respir Cell Mol Biol*. (2011) 45(4):740-5.<br>20. Shalova IN, Kajiji T, Lim JY, Gomez-Pina V, Fernandez-Ruiz I, Arnalich F, *et al*. CD16 regulates TRIF-dependent TLR4 response in human monocytes and their subsets. *J Immunol*. (2012) 188(8):3584-93.<br>21. Sumpter TL, Packiam V, Turnquist HR, Castellaneta A, Yoshida O, Thomson AW. DAP12 promotes IRAK-M expression and IL-10 production by liver myeloid dendritic cells and restrains their T cell allostimulatory ability. *J Immunol*. (2011) 186(4):1970-80.<br>22. Taylor AW. The immunomodulating neuropeptide alpha-melanocyte-stimulating hormone (α-MSH) suppresses LPS-stimulated TLR4 with IRAK-M in macrophages. *J Neuroimmunol*. (2005) 162(1-2):43-50.<br>23. Woehrle T, Du W, Goetz A, Hsu H-Y, Joos TO, Weiss M, *et al*. Pathogen specific cytokine release reveals an effect of TLR2 Arg753Gln during *Candida* sepsis in humans. *Cytokine*. (2008) 41(3):322-9.<br>24. Yang Q, Calvano SE, Lowry SF, Androulakis IP. A dual negative regulation model of Toll-like receptor 4 signaling for endotoxin preconditioning in human endotoxemia. *Math Biosci*. (2011) 232(2):151-63.<br>25. Zhang FX, Kirschning CJ, Mancinelli R, Xu XP, Jin Y, Faure E, *et al*. Bacterial lipopolysaccharide activates nuclear factor-κB through interleukin-1 signaling mediators in cultured human dermal endothelial cells and mononuclear phagocytes. *J Biol Chem*. (1999) 274(12):7611-4.
In vitro studies	1. Geng S, Chen K, Yuan R, Peng L, Maitra U, Diao N, et al. The persistence of low-grade inflammatory monocytes contributes to aggravated atherosclerosis. *Nat commun*. 2016;7:13436.<br>2. Gunthner R, Kumar VR, Lorenz G, Anders HJ, Lech M. Pattern-recognition receptor signaling regulator mRNA expression in humans and mice, and in transient inflammation or progressive fibrosis. *Int J Mol Sci*. 2013;14(9):18124-47.<br>3. Sanaei R, Rezaei N, Aghamohammadi A, Delbandi AA, Teimourian S, Yazdani R, et al. Evaluation of the TLR negative regulatory network in CVID patients. *Genes Immun*. 2018;20(3):1-9.<br>4. Sung NY, Yang MS, Song DS, Kim JK, Park JH, Song BS, et al. Procyanidin dimer B2-mediated IRAK-M induction negatively regulates TLR4 signaling in macrophages. *Biochem Biophys Res Commun*. 2013;438(1):122-8.<br>5. Sanaei R, Rezaei N, Aghamohammadi A, Delbandi A-A, Tavasolian P, Tajik N. Disturbed transcription of TLRs' negative regulators and cytokines secretion among TLR4-and 9-activated PBMCs of agammaglobulinemic patients. *Immunol Invest*. 2019;48(8):860-74.<br>6. Adib-Conquy M, Cavaillon JM. Gamma interferon (IFNγ) and granulocyte/monocyte colony-stimulating factor (GM-CSF) prevent endotoxin tolerance in human monocytes by promoting interleukin-1 receptor-associated kinase expression and its association to MyD88 and not by modulating TLR4 expression. *J Biol Chem*. 2002;277(31):27927-34.<br>7. Al Mubarak R, Roberts N, Mason RJ, Alper S, Chu HW. Comparison of pro-and anti-inflammatory responses in paired human primary airway epithelial cells and alveolar macrophages. *Respir Res*. 2018;19(1):126.<br>8. Al-Shaghdali K, Durante B, Hayward C, Beal J, Foey A. Macrophage subsets exhibit distinct *E. coli*-LPS tolerisable cytokines associated with the negative regulators, IRAK-M and Tollip. *PLos One*. 2019;14(5):e0214681.<br>9. Almeida AS, Lago PM, Boechat N, Huard RC, Lazzarini LC, Santos AR, et al. Tuberculosis is associated with a down-modulatory lung immune response that impairs Th1-type immunity. *J Immunol*. 2009;183(1):718-31.<br>10. Ara T, Kurata K, Hirai K, Uchihashi T, Uematsu T, Imamura Y, et al. Human gingival fibroblasts are critical in sustaining inflammation in periodontal disease. *J Periodontal Res*. 2009;44(1):21-7.<br>11. Brudecki L, Ferguson DA, McCall CE, El Gazzar M. Mitogen-activated protein kinase phosphatase 1 disrupts proinflammatory protein synthesis in endotoxin-adapted monocytes. *Clin Vaccine Immunol*. 2013;20(9):1396-404.

12. Chen H, Cowan MJ, Hasday JD, Vogel SN, Medvedev AE. Tobacco smoking inhibits expression of proinflammatory cytokines and activation of IL-1R-associated kinase, p38, and NF-κB in alveolar macrophages stimulated with TLR2 and TLR4 agonists. *J Immunol*. 2007;179(9):6097-106.
13. Chen W, Saxena A, Li N, Sun J, Gupta A, Lee DW, et al. Endogenous IRAK-M attenuates postinfarction remodeling through effects on macrophages and fibroblasts. *Arterioscler Thromb Vasc Biol*. 2012;32(11):2598-608.
14. Cole TS, Zhang M, Standiford TJ, Newstead M, Luther J, Zhang J, et al. IRAK-M modulates expression of IL-10 and cell surface markers CD80 and MHC II after bacterial re-stimulation of tolerized dendritic cells. *Immunol Lett*. 2012;144(1-2):49-59.
15. Daskalaki MG, Vyrla D, Harizani M, Doxaki C, Eliopoulos AG, Roussis V, et al. Neorogioltriol and related diterpenes from the red alga *Laurencia* inhibit inflammatory bowel disease in mice by suppressing M1 and promoting M2-like macrophage responses. *Mar Drugs*. 2019;17(2).
16. del Fresno C, Gómez-García L, Caveda L, Escoll P, Arnalich F, Zamora R, et al. Nitric oxide activates the expression of IRAK-M via the release of TNF-α in human monocytes. *Nitric Oxide: Biol Chem*. 2004;10(4):213-20.
17. del Fresno C, Otero K, Gomez-Garcia L, Gonzalez-Leon MC, Soler-Ranger L, Fuentes-Prior P, et al. Tumor cells deactivate human monocytes by up-regulating IL-1 receptor associated kinase-M expression via CD44 and TLR4. *J Immunol*. 2005;174(5):3032-40.
18. del Fresno C, Soler-Rangel L, Soares-Schanoski A, Gomez-Pina V, Gonzalez-Leon MC, Gomez-Garcia L, et al. Inflammatory responses associated with acute coronary syndrome up-regulate IRAK-M and induce endotoxin tolerance in circulating monocytes. *J Endotoxin Res*. 2007;13(1):39-52.
19. Domon H, Honda T, Oda T, Yoshie H, Yamazaki K. Early and preferential induction of IL-1 receptor-associated kinase-M in THP-1 cells by LPS derived from *Porphyromonas gingivalis*. *J Leukoc Biol*. 2008;83(3):672-9.
20. Du J, Nicolaes GA, Kruijswijk D, Versloot M, van der Poll T, van 't Veer C. The structure function of the death domain of human IRAK-M. *Cell Commun Signal*. 2014;12:77.
21. Escoll P, del Fresno C, Garcia L, Valles G, Lendinez MJ, Arnalich F, et al. Rapid up-regulation of IRAK-M expression following a second endotoxin challenge in human monocytes and in monocytes isolated from septic patients. *Biochem Biophys Res Commun*. 2003;311(2):465-72.
22. Ferlito M, Romanenko OG, Ashton S, Squadrito F, Halushka PV, Cook JA. Effect of cross-tolerance between endotoxin and TNF-α or IL-1β on cellular signaling and mediator production. *J Leukoc Biol*. 2001;70(5):821-9.
23. Fernandez-Ruiz I, Arnalich F, Cubillos-Zapata C, Hernandez-Jimenez E, Moreno-Gonzalez R, Toledano V, et al. Mitochondrial DAMPs induce endotoxin tolerance in human monocytes: an observation in patients with myocardial infarction. *PLos One*. 2014;9(5):e95073.
24. Finamore A, Roselli M, Imbinto A, Seeboth J, Oswald IP, Mengheri E. *Lactobacillus amylovorus* inhibits the TLR4 inflammatory signaling triggered by enterotoxigenic *Escherichia coli* via modulation of the negative regulators and involvement of TLR2 in intestinal Caco-2 cells and pig explants. *PLos One*. 2014;9(4):e94891.
25. Hajishengallis G, Sojar H, Genco RJ, DeNardin E. Intracellular signaling and cytokine induction upon interactions of *Porphyromonas gingivalis* fimbriae with pattern-recognition receptors. *Immunol Invest*. 2004;33(2):157-72.
26. Harada K, Isse K, Sato Y, Ozaki S, Nakanuma Y. Endotoxin tolerance in human intrahepatic biliary epithelial cells is induced by upregulation of IRAK-M. *Liver Int*. 2006;26(8):935-42.
27. Hassan F, Islam S, Tumurkhuu G, Dagvadorj J, Naiki Y, Komatsu T, et al. Involvement of interleukin-1 receptor-associated kinase (IRAK)-M in toll-like receptor (TLR) 7-mediated tolerance in RAW 264.7 macrophage-like cells. *Cell Immunol*. 2009;256(1-2):99-103.
28. Im J, Baik JE, Kim KW, Kang SS, Jeon JH, Park OJ, et al. *Enterococcus faecalis* lipoteichoic acid suppresses *Aggregatibacter actinomycetemcomitans* lipopolysaccharide-induced IL-8 expression in human periodontal ligament cells. *Int Immunol*. 2015;27(8):381-91.
29. Jiang D, Matsuda J, Berman R, Schaefer N, Stevenson C, Gross J, et al. A novel mouse model of conditional IRAK-M deficiency in myeloid cells: application in lung *Pseudomonas aeruginosa* infection. *Innate Immun*. 2017;23(2):206-15.
30. Julian MW, Strange HR, Ballinger MN, Hotchkiss RS, Papenfuss TL, Crouser ED. Tolerance and cross-tolerance following toll- like receptor (TLR)-4 and -9 activation are mediated by IRAK-M and modulated by IL-7 in murine splenocytes. *PLos One*. 2015;10(7):e0132921.

31. Kanakaraj P, Schafer PH, Cavender DE, Wu Y, Ngo K, Grealish PF, et al. Interleukin (IL)-1 receptor-associated kinase (IRAK) requirement for optimal induction of multiple IL-1 signaling pathways and IL-6 production. *J Exp Med*. 1998;187(12):2073-9.
32. Kim CH, Kim GH, Kim JY, Kim NR, Jung BJ, Jeong JH, et al. Probiotic genomic DNA reduces the production of pro-inflammatory cytokine tumor necrosis factor-alpha (TNF-α). *FEMS Microbiol Lett*. 2012;328(1):13-9.
33. Kobayashi K, Hernandez LD, Galan JE, Janeway CA, Medzhitov R, Flavell RA. IRAK-M is a negative regulator of toll-like receptor signaling. *Cell*. 2002;110(2):191-202.
34. Lagler H, Sharif O, Haslinger I, Matt U, Stich K, Furtner T, et al. TREM-1 activation alters the dynamics of pulmonary IRAK-M expression *in vivo* and improves host defense during *Pneumococcal Pneumonia*. *J Immunol*. 2009;183(3):2027-36.
35. Lee A, Qiao Y, Grigoriev G, Chen J, Park-Min KH, Park SH, et al. Tumor necrosis factor α induces sustained signaling and a prolonged and unremitting inflammatory response in rheumatoid arthritis synovial fibroblasts. *Arthritis Rheum*. 2013;65(4):928-38.
36. Li Y, Annette EH, Lagoo AS, Kuchibhatla M, Pan H, Cohen HJ, et al. Differential gene expression of interleukin-1 receptor associated kinase-1 and interleukin-1 receptor associated kinase-M in peripheral blood mononuclear cells of young and aged rats following preconditioning with endotoxin. *Shock*. 2009;31(1):55-63.
37. Liu X, Qin Y, Dai A, Zhang Y, Xue H, Ni H, et al. SMAD4 is involved in the development of endotoxin tolerance in microglia. *Cell Mol Neurobiol*. 2016;36(5):777-88.
38. Liu ZJ, Yan LN, Li XH, Xu FL, Chen XF, You HB, et al. Up-regulation of IRAK-M is essential for endotoxin tolerance induced by a low dose of lipopolysaccharide in Kupffer cells. *J Surg Res*. 2008;150(1):34-9.
39. Lunz JG, III, Specht SA, Murase N, Isse K, Demetris AJ. Gut-derived commensal bacterial products inhibit liver dendritic cell maturation by stimulating hepatic interleukin-6/signal transducer and activator of transcription 3 activity. *Hepatology*. 2007;46(6):1946-59.
40. Lyroni K, Patsalos A, Daskalaki MG, Doxaki C, Soennichsen B, Helms M, et al. Epigenetic and transcriptional regulation of IRAK-M expression in macrophages. *J Immunol*. 2017;198(3):1297-307.
41. Maldifassi MC, Atienza G, Arnalich F, López-Collazo E, Cedillo JL, Martín-Sánchez C, et al. A new IRAK-M-mediated mechanism implicated in the anti-inflammatory effect of nicotine via α7 nicotinic receptors in human macrophages. *PLos One*. 2014;9(9):e108397.
42. Mandrekar P, Bala S, Catalano D, Kodys K, Szabo G. The opposite effects of acute and chronic alcohol on lipopolysaccharide-induced inflammation are linked to IRAK-M in human monocytes. *J Immunol*. 2009;183(2):1320-7.
43. Nakayama K, Okugawa S, Yanagimoto S, Kitazawa T, Tsukada K, Kawada M, et al. Involvement of IRAK-M in peptidoglycan-induced tolerance in macrophages. *J Biol Chem*. 2004;279(8):6629-34.
44. Nguyen HA, Rajaram MV, Meyer DA, Schlesinger LS. Pulmonary surfactant protein A and surfactant lipids upregulate IRAK-M, a negative regulator of TLR-mediated inflammation in human macrophages. *Am J Physiol Lung Cell Mol Physiol*. 2012;303(7):L608-16.
45. Odoms K, Shanley TP, Wong HR. Short-term modulation of interleukin-1β signaling by hyperoxia: uncoupling of IκB kinase activation and NF-κB-dependent gene expression. *Am J Physiol Lung Cell Mol Physiol*. 2004;286(3 30-3):L554-L62.
46. Parmar N, Chandrakar P, Vishwakarma P, Singh K, Mitra K, Kar S. *Leishmania donovani* exploits tollip, a multitasking protein, to impair TLR/IL-1R signaling for its survival in the host. J Immunol. 2018;201(3):957-70.
47. Peck OM, Williams DL, Breuel KF, Kalbfleisch JH, Fan H, Tempel GE, et al. Differential regulation of cytokine and chemokine production in lipopolysaccharide-induced tolerance and priming. *Cytokine*. 2004;26(5):202-8.
48. Petricevic B, Wessner B, Sachet M, Vrbanec D, Spittler A, Bergmann M. CL097, a TLR7/8 Ligand, inhibits TLR-4-dependent activation of IRAK-M and BCL-3 expression. *Shock*. 2009;32(5):484-90.
49. Rajaiah R, Perkins DJ, Polumuri SK, Zhao A, Keegan AD, Vogel SN. Dissociation of endotoxin tolerance and differentiation of alternatively activated macrophages. *J Immunol*. 2013;190(9):4763-72.

50. Saito K, Katakura K, Suzuki R, Suzuki T, Ohira H. Modulating Toll-like receptor 4 signaling pathway protects mice from experimental colitis. *Fukushima J Med Sci*. 2013;59(2):81-8.

51. Scotton CJ, Martinez FO, Smelt MJ, Sironi M, Locati M, Mantovani A, et al. Transcriptional profiling reveals complex regulation of the monocyte IL-1β system by IL-13. *J Immunol*. 2005;174(2):834-45.

52. Shen W, Stone K, Jales A, Leitenberg D, Ladisch S. Inhibition of TLR activation and up-regulation of IL-1R-associated kinase-M expression by exogenous gangliosides. *J Immunol*. 2008;180(7):4425-32.

53. Shiu J, Czinn SJ, Kobayashi KS, Sun Y, Blanchard TG. IRAK-M expression limits dendritic cell activation and proinflammatory cytokine production in response to *Helicobacter pylori*. *PLos One*. 2013;8(6):e66914.

54. Soares-Schanoski A, Gomez-Pina V, del Fresno C, Rodriguez-Rojas A, Garcia F, Glaria A, et al. 6-Methylprednisolone down-regulates IRAK-M in human and murine osteoclasts and boosts bone-resorbing activity: a putative mechanism for corticoid-induced osteoporosis. *J Leukoc Biol*. 2007;82(3):700-9.

55. Srivastav S, Saha A, Barua J, Ukil A, Das PK. IRAK-M regulates the inhibition of TLR-mediated macrophage immune response during late *in vitro Leishmania donovani* infection. *Eur J Immunol*. 2015;45(10):2787-97.

56. Standiford TJ, Kuick R, Bhan U, Chen J, Newstead M, Keshamouni VG. TGF-β-induced IRAK-M expression in tumor-associated macrophages regulates lung tumor growth. *Oncogene*. 2011;30(21):2475-84.

57. Stark RJ, Choi H, Koch SR, Fensterheim BA, Lamb FS, Sherwood ER. Endothelial cell tolerance to lipopolysaccharide challenge is induced by monophosphoryl lipid A. *Clin Sci*. 2016;130(6):451-61.

58. Stiehm M, Peters K, Wiesmüller KH, Bufe A, Peters M. A novel synthetic lipopeptide is allergy-protective by the induction of LPS-tolerance. *Clin Exp Allergy*. 2013;43(7):785-97.

59. Su J, Xie Q, Wilson I, Li L. Differential regulation and role of interleukin-1 receptor associated kinase-M in innate immunity signaling. *Cell Signal*. 2007;19(7):1596-601.

60. Su J, Zhang T, Tyson J, Li L. The interleukin-1 receptor-associated kinase-M selectively inhibits the alternative, instead of the classical NF-κB pathway. *J Innate Immun*. 2009;1(2):164-74.

61. Sun Y, Li H, Sun MJ, Zheng YY, Gong DJ, Xu Y. Endotoxin tolerance induced by lipopolysaccharides derived from *Porphyromonas gingivalis* and *Escherichia coli*: Alternations in toll-like receptor 2 and 4 signaling pathway. *Inflammation.* 2014;37(1):268-76.

62. Swantek JL, Tsen MF, Cobb MH, Thomas JA. IL-1 receptor-associated kinase modulates host responsiveness to endotoxin. *J Immunol*. 2000;164(8):4301-6.

63. Tazi KA, Quioc JJ, Saada V, Bezeaud A, Lebrec D, Moreau R. Upregulation of TNF-α production signaling pathways in monocytes from patients with advanced cirrhosis: Possible role of Akt and IRAK-M. *J Hepatol*. 2006;45(2):280-9.

64. Tiwari RL, Singh V, Singh A, Barthwal MK. IL-1R-associated kinase-1 mediates protein kinase C-delta (PKC-δ) - induced IL-1β production in monocytes. *J Immunol*. 2011;187(5):2632-45.

65. Turrel-Davin F, Cazalis MA, Venet F, Pachot A, Alberti-Segui C, Lepape A, et al. mRNA-based approach to monitor recombinant gamma-interferon (IFN-γ) restoration of LPS-induced endotoxin tolerance. *Intensive Care Med*. 2011;1):S116.

66. Wesche H, Gao X, Li X, Kirschning CJ, Stark GR, Cao Z. IRAK-M is a novel member of the pelle/interleukin-1 receptor-associated kinase (IRAK) family. *J Biol Chem*. 1999;274(27):19403-10.

67. Wiersinga WJ, van't Veer C, van den Pangaart PS, Dondorp AM, Day NP, Peacock SJ, et al. Immunosuppression associated with interleukin-1R-associated-kinase-M upregulation predicts mortality in Gram-negative sepsis (melioidosis). *Crit Care Med*. 2009;37(2):569-76.

68. Wu Q, Jiang D, Smith S, Thaikoottathil J, Martin RJ, Bowler RP, et al. IL-13 dampens human airway epithelial innate immunity through induction of IL-1 receptor-associated kinase M. *J Allergy Clin Immunol*. 2012;129(3):825-U330.

69. Xiong Y, Pennini M, Vogel SN, Medvedev AE. IRAK4 kinase activity is not required for induction of endotoxin tolerance but contributes to TLR2-mediated tolerance. *J Leukoc Biol*. 2013;94(2):291-300.
70. Xiong Y, Qiu F, Piao W, Song C, Wahl LM, Medvedev AE. Endotoxin tolerance impairs IL-1 receptor-associated kinase (IRAK) 4 and TGF-β-activated kinase 1 activation, K63-linked polyubiquitination and assembly of IRAK1, TNF receptor-associated factor 6, and IκB kinase-γ and increases A20 expression. *J Biol Chem*. 2011;286(10):7905-16.
71. Zhang Y, Hou C, Yu S, Xiao J, Zhang Z, Zhai Q, et al. IRAK-M in macrophages around septically and aseptically loosened hip implants. *J Biomed Mater Res A*. 2012;100A(1):261-8.
72. Zhang Y, Yu S, Xiao J, Hou C, Li Z, Zhang Z, et al. Wear particles promote endotoxin tolerance in macrophages by inducing interleukin-1 receptor-associated kinase-M expression. *J Biomed Mater Res A*. 2013;101(3):733-9.
73. Zhou H, Yu M, Fukuda K, Im J, Yao P, Cui W, et al. IRAK-M mediates Toll-like receptor/IL-1R-induced NF-κB activation and cytokine production. *EMBO J*. 2013;32(4):583-96.
74. Zhou H, Yu M, Zhao J, Martin BN, Roychowdhury S, McMullen MR, et al. IRAK-M-mincle axis links cell death to inflammation: Pathophysiological implications for chronic alcoholic liver disease. *Hepatology*. 2016;64(6):1978-93.
75. Zhou Y, Xia Q, Wang X, Fu S. Endotoxin tolerant dendritic cells suppress inflammatory responses in splenocytes via interleukin-1 receptor associated kinase (IRAK)-M and programmed death-ligand 1 (PDL-1). *Med Sci Mon Int Med J Exp Clin Res*. 2018;24:4798.
