# Supplementary material for: A systematic review and meta-analyses of interleukin-1 receptor associated kinase 3 (IRAK3) action on inflammation in in vivo models for the study of sepsis
Source: PLoS One. 2022 Feb 15;17(2):e0263968. doi: 10.1371/journal.pone.0263968 (PMC8846508; doi:10.1371/journal.pone.0263968)
Supplement: S3 Table — (PDF) [file pone.0263968.s005.pdf]

S3 Table. Summary of risk of bias in the included animal *in vivo* studies.

Article	1: Was the allocation sequence adequately generated and applied?	2: Were the groups similar at baseline or were they adjusted for confounders in the analysis?	3: Was the allocation adequately concealed?	4: Were the animals randomly housed during the experiment?	5: Were the caregivers and/or investigators blinded from knowledge which intervention each animal received during the experiment?	6: Were animals selected at random for outcome assessment?	7: Was the outcome assessor blinded?	8: Were incomplete outcome data adequately addressed?	9: Are reports of the study free of selective outcome reporting?	10: Was the study apparently free of other problems that could result in high risk of bias?
Aboyoussef *et al* (2021)	Unclear	Yes	Unclear	Unclear	Unclear	Unclear	Unclear	Yes	Yes	Yes
Berglund *et al*. (2010)	Unclear	Yes	Unclear	Unclear	Unclear	Unclear	Yes	Yes	Yes	Yes
Deng *et al*. (2006)	Unclear	Yes	Unclear	Unclear	Unclear	Unclear	Unclear	Unclear	Yes	Yes
Funahashi *et al*. (2019)	Unclear	Unclear	Unclear	Unclear	Unclear	Unclear	Unclear	Yes	Yes	Yes
Gandhirajan *et al.* (2021)	Unclear	Yes	Unclear	Unclear	Unclear	Unclear	Unclear	Unclear	Yes	Yes
Gong *et al*. (2017)	Unclear	Yes	Unclear	Unclear	Unclear	Unclear	Unclear	Yes	Yes	Yes
Gribar *et al*. (2009)	Unclear	Yes	Unclear	Unclear	Unclear	Unclear	Yes	Unclear	Yes	Yes
Han *et al*. (2008)	Unclear	Yes	Unclear	Unclear	Unclear	Unclear	Unclear	Yes	Yes	Yes
Hayashi *et al*. (2009)	Unclear	Yes	Unclear	Unclear	Unclear	Unclear	Unclear	Yes	Yes	Yes
Hoogerwerf *et al*. (2012)	Unclear	Unclear	Unclear	Unclear	Unclear	Unclear	Unclear	Unclear	Yes	Yes
Kallapur *et al*. (2007)	Unclear	Yes	Unclear	Yes	Unclear	Unclear	Unclear	Unclear	Yes	Yes
Kim *et al*. (2008)	Unclear	Yes	Unclear	Unclear	Unclear	Unclear	Unclear	Yes	Yes	Yes

Kingsley *et al*. (2020)	Unclear	Yes	Unclear	Unclear	Unclear	Unclear	Unclear	Yes	Yes	Yes
Krogmann *et al*. (2020)	Unclear	Yes	Unclear	Unclear	Unclear	Unclear	Unclear	Yes	Yes	Yes
Li *et al*. (2013)	Unclear	Yes	Unclear	Unclear	Unclear	Unclear	Unclear	Yes	Yes	Yes
Liu et al. (2016)	Unclear	Yes	Unclear	Unclear	Unclear	Unclear	Unclear	Yes	Yes	Yes
Lyn-Kew *et al*. (2010)	Unclear	Yes	Unclear	Unclear	Unclear	Unclear	Unclear	Yes	Yes	Yes
Miyata *et al*. (2015)	Unclear	Yes	Unclear	Unclear	Unclear	Unclear	Unclear	Yes	Yes	Yes
Paik *et al*. (2019)	Unclear	Yes	Unclear	Unclear	Unclear	Unclear	Unclear	Yes	Yes	Yes
Petzl *et al*. (2012)	Unclear	Yes	Unclear	Unclear	Unclear	Unclear	Unclear	Unclear	Yes	Yes
Rothschild *et al*. (2017)	Unclear	Yes	Unclear	Unclear	Yes	Unclear	Yes	Yes	Yes	Yes
Shao *et al*. (2011)	Unclear	Yes	Unclear	Unclear	Unclear	Unclear	Unclear	Unclear	Yes	Yes
Szatanik *et al*. (2011)	Unclear	Yes	Unclear	Unclear	Unclear	Unclear	Unclear	Yes	Yes	Yes
Tan *et al*. (2019)	Unclear	Unclear	Unclear	Unclear	Unclear	Unclear	Unclear	Yes	Yes	Yes
Takebayashi *et al*. (2009)	Unclear	Yes	Unclear	Unclear	Unclear	Unclear	Unclear	Yes	Yes	Yes
Tseng *et al*. (2020)	Unclear	Yes	Unclear	Yes	Unclear	Unclear	Unclear	Yes	Yes	Yes
van der Windt *et al*. (2012)	Unclear	Yes	Unclear	Unclear	Unclear	Unclear	Unclear	Unclear	Yes	Yes
Xia *et al*. (2018)	Unclear	Yes	Unclear	Unclear	Unclear	Unclear	Unclear	Yes	Yes	Yes
Xie *et al*. (2019)	Unclear	Yes	Unclear	Yes	Unclear	Unclear	Unclear	Yes	Yes	Yes
Xiong *et al*. (2011)	Unclear	Yes	Unclear	Unclear	Unclear	Unclear	Unclear	Unclear	Yes	Yes

Yang and Zhao (2021)	Unclear	Yes	Unclear	Yes	Yes	Unclear	Unclear	Yes	Yes	Yes
Yee and Hamerman (2013)	Unclear	Yes	Unclear	Unclear	Unclear	Unclear	Unclear	Unclear	Yes	Yes
Yu *et al*. (2017)	Unclear	Yes	Unclear	Yes	Unclear	Unclear	Unclear	Yes	Yes	Yes
Zhang *et al*. (2020)	Unclear	Yes	Unclear	Yes	Unclear	Unclear	Unclear	Yes	Yes	Yes
Zhang and Wang (2021)	Unclear	Yes	Unclear	Unclear	Unclear	Unclear	Unclear	Yes	Yes	Yes
Zhao *et al*. (2019)	Unclear	Yes	Unclear	Unclear	Unclear	Unclear	Unclear	Unclear	Yes	Yes
Zhao *et al*. (2021)	Unclear	Yes	Unclear	Unclear	Unclear	Unclear	Unclear	Yes	Yes	Yes
